# Epidemiology of Tularemia among Humans and Animals, Baden-Wuerttemberg, Germany, 2012–2022

**DOI:** 10.3201/eid3104.240414

**Published:** 2025-04

**Authors:** Sabrina Nothdurfter, Jörg Linde, Reinhard Sting, Herbert Tomaso, Klaus Heuner, Maylin Meincke, Stefan O. Brockmann, Christiane Wagner-Wiening

**Affiliations:** ECDC Fellowship Programme, Field Epidemiology Path, European Centre for Disease Prevention and Control, Stockholm, Sweden (S. Nothdurfter); Baden-Wuerttemberg State Health Office, Ministry of Social Affairs, Health and Integration, Stuttgart, Germany (S. Nothdurfter, M. Meincke, S.O. Brockmann, C. Wagner-Wiening); Institute of Bacterial Infections and Zoonoses, Friedrich-Loeffler-Institute, Jena, Germany (J. Linde, H. Tomaso); Chemical and Veterinary Analysis Agency Stuttgart, Fellbach, Germany (R. Sting); Highly Pathogenic Microorganisms (ZBS 2), Centre for Biological Threats and Special Pathogens, Robert Koch Institute, Berlin, Germany (K. Heuner)

**Keywords:** tularemia, *Francisella tularensis*, bacteria, zoonoses, One Health, whole-genome sequencing, Germany, antimicrobial resistance

## Abstract

Tularemia, a zoonosis caused by *Francisella tularensis*, is endemic in Baden-Wuerttemberg, Germany. To determine tularemia epidemiology in this region, we characterized the genetic diversity of *F. tularensis* in human and animal isolates during 2012–2022 by using whole-genome sequencing, combined with human and veterinary surveillance data analysis. Human case numbers varied; most cases occurred in 2021 (n = 34). Arthropod bites were reported most in cases with information on animal exposure (45%, n = 43). Poisson regression confirmed a significant increase in human cases during the study period (p<0.001). No seasonal pattern was identified, but case numbers were lowest in winter. Human surveillance data often lacked exposure details. Positivity rates in animals were 5%–34%, increasing since 2017. Human isolates often clustered with hare-derived strains, although transmission routes often remain unclear. These findings emphasize the importance of combining genome sequencing with detailed epidemiologic tracing to identify infection sources and improve surveillance data.

Tularemia is a zoonotic disease caused by the bacterium *Francisella tularensis*, prevalent in the northern hemisphere (*1*). In Europe, most cases are caused by *F. tularensis* subspecies *holarctica*. In Germany, tularemia is notifiable in humans and animals (*2*,*3*), and cases are reported in almost every state (*4*,*5*). Baden-Wuerttemberg is among the states with the highest incidence of human tularemia; 3.1 cases/1 million population were reported in 2021, surpassing the national rate of 1.4 cases/1 million population (*5*–*6*). In Baden-Wuerttemberg, the disease is endemic in animals, particularly affecting brown hares (*Lepus europaeus*), which might play a major role in transmitting tularemia to humans (*7*).

Tularemia can be transmitted to humans by various animals. Arthropods such as ticks, tabanids, and mosquitos might serve as vectors, and small mammals might serve as reservoirs, varying by region. Moreover, transmission can occur through consumption of or contact with contaminated food or water and through inhalation of contaminated aerosols (*8*–*12*). The transmission route and entry point of the infection determine incubation period, clinical form, and manifestations, which often include nonspecific signs and symptoms (*8*,*9*); fever and swollen lymph nodes are the most commonly reported (*4*). Tularemia has varying forms, including ulceroglandular, oropharyngeal, or pneumonic tularemia (*13*).

Recent whole-genome sequencing (WGS) of *F. tularensis* isolates from humans and animals has improved our understanding of the phylogeography and molecular epidemiology of this pathogen. For *F. tularensis*, canonical single-nucleotide polymorphisms (SNPs) identified through WGS have been established. Canonical SNPs define loci of ancestral strains, which can mutate and lead to a new branch of derived strains. Those canonical SNPs and the resulting major clades and subclades were given names on the basis of a combination of letters and numbers (*14*). In Germany, major clade B.6, corresponding to biovar I (sensitive to erythromycin), is more frequent in the southwest, whereas major clade B.12, or biovar II (resistant to erythromycin), is more prevalent in the northeast. Analysis of the distribution of subclades indicates that isolates belonging to the same canonical SNP type might circulate in both humans and animals (*4*,*15*,*16*). Of note, small geographic regions were found to be inhabited by bacterial strains that can be assigned to distant clades and even the other biovar. Studies in Sweden and Germany found that near–genetically identical isolates of the same subclades can be found dispersed across larger distances (*15*,*17*,*18*).

This research provides an overview of the epidemiology of tularemia in Baden-Wuerttemberg, linking human and veterinary surveillance with genome data. Because *F. tularensis* is a potential biologic agent and tularemia is endemic to Europe (*19*), knowing which strains are circulating in the animal and human population in respective regions is key. Strains of other subspecies or unexpected genotypes should alert the health system and prompt further investigations (*20*). This study examines human transmission modes, regional distribution, and genomic similarities and differences between human and animal genotypes. Identifying regional clusters and addressing potential underreporting are crucial for improving public health measures and raising awareness.

## Methods and Materials

### Surveillance Data

We analyzed surveillance data from human cases reported in Baden-Wuerttemberg during January 2012–December 2022. We included cases meeting the reference definition for tularemia in Germany (*21*) and excluded cases in persons with travel history and known risk exposures outside of Baden-Wuerttemberg.

The state veterinary authorities (Veterinary Analysis Agencies) in Baden-Wuerttemberg provided animal data from passive surveillance, primarily consisting of animal carcasses found by hunters, farmers, and hikers (*7*). We included all animals with available laboratory results for *F. tularensis* within the observation period.

We described human case characteristics and stratified human cases by meteorological seasons and years for temporal analysis with Poisson regression and calculated median time lag of symptom onset to reporting. For cases with missing onset dates (n = 17 [11%]), we estimated symptom onset by subtracting the median 29-day lag from the notification date. We used sample dates to calculate positivity rates in animal cases and stratified human and animal cases by districts. We used R version 4.1.2 (The R Project for Statistical Computing, https://www.r-project.org) for statistical analysis.

### Bacterial Identification, Genome Sequencing, and Data Analysis

Animals identified through passive surveillance were tested for *F. tularensis* at the Veterinary Analysis Agencies. The isolates were further characterized at the Institute of Bacterial Infections and Zoonoses, Federal Research Institute for Animal Health (Jena, Germany). Cultivation, DNA extraction, and Illumina sequencing of 47 human and animal samples were performed as previously described (*4*,*15*,*16*).

To compare the genomes of strains collected within this study with previously published genomes from Baden-Wuerttemberg, we downloaded raw sequencing data of 7 human (*16*) and 61 animal (*15*) strains (Appendix Figure) and analyzed them with the WGSBAC pipeline version 2.2.3 (*15*). We controlled raw sequencing quality using FastQC version 0.11.7 (https://www.bioinformatics.babraham.ac.uk/projects/fastqc) and calculated raw coverage and performed assembly of raw reads using Shovill version 1.0.4 (https://github.com/tseemann/shovill). We checked the quality of assemblies by using QUAST version 5.0.2 (*22*). We used Kraken2 version 2.1.1 to test for possible contamination (*23*). To detect subspecies-specific marker genes, we used in_silico_PCR version 0.5.1 (https://github.com/egonozer/in_silico_pcr) to search for the *F. tularensis* chromosomal marker RD-1 and checked the amplicon size for the specific length (924 bp) of *F. tularensis* subsp. *holarctica*.

We performed genotyping on the basis of predefined canonical SNPs with CanSNPer (*24*) and CanSNPer2 (https://github.com/FOI-Bioinformatics/CanSNPer2). In addition, mapping-based SNP typing with OSU18 as reference was performed with Snippy version 4.3.6 (https://github.com/tseemann/snippy), which also constructed a core-genome SNP (cgSNP) alignment of all strains. We used Snps-dists version 0.63 (https://github.com/tseemann/snp-dists) to calculate the pairwise SNP distances between strains. Using the cgSNP distance matrix, we performed clustering with the hierClust function version 5.1 of R (https://www.rdocumentation.org/packages/momr/versions/1.1/topics/hierClust). We used a cutoff of 5 cgSNPs to define genomically highly similar strains (*15*). We constructed phylogenetic trees with RAxML version 8 (*25*) and visualized with the interactive Tree of Life (iTOL) version 4 web tool (*26*).

### Ethics Statement

Ethical approval was not required because no personal identifying information was processed. Tularemia is a notifiable disease, and the analysis of routinely collected surveillance and sequencing data are part of the health authorities’ responsibilities under the Protection Against Infection Act (German: Infektionsschutzgesetz).

## Results

### Epidemiology of Notified Human Cases

In Baden-Wuerttemberg, 152 tularemia infections, including 5 deaths, were reported during 2012–2022. Annual cases ranged from 3 to 34 (median 10); annual incidence rates were 0.27–3.1/1 million population. Most cases (n = 34) were reported in 2021, representing a 3-fold increase of cases over the previous year (n = 11). The median lag time from symptom onset to reporting was 29 days; 18 cases were reported after >96 days.

Of the 152 cases, 106 (70%) were in men and 46 (30%) in women (Table 1); the median patient age was 53 years (interquartile range 37–65 years). More than half of patients (66%, n = 85) were hospitalized. The most common laboratory confirmation was serology (67%, n = 102) followed by PCR assay only (22%, n = 33) and PCR plus culture (20%, n = 31). Symptoms were reported in all but 1 case; fever (75%, n = 113) and lymph node swelling (69%, n = 104) were most common, followed by skin ulcer (24%, n = 36), pneumonia (15%, n = 23), and dyspnea (11%, n = 17). The main forms of tularemia were glandular (35%, n = 53) and ulceroglandular (23%, n = 35). Pneumonic tularemia occurred in 17% (n = 26) of the cases, mostly reported in 2019 and 2022 (n = 18); 8 cases occurred in 2021. Most cases with dyspnea (n = 12) were reported during 2019–2022; 9 of those cases were reported in 2021 and 2022. Less than 10% of cases showed symptoms of oropharyngeal tularemia; 12% of cases (n = 18) had unspecific symptoms and unknown exposure risk, possibly indicating typhoidal tularemia.

**Table 1:** Characteristics of notified tularemia human cases based on surveillance data in study of epidemiology of tularemia among humans and animals, Baden-Wuerttemberg, Germany, 2012–2022*

Variable	Value	
Sex, n = 152		
M	106 (70)	
F	46 (30)	
Age group, y, n = 152		
0–9	8 (5)	
10–19	10 (7)	
20–29	12 (8)	
30–39	15 (10)	
40–49	22 (14)	
50–59	31 (20)	
60–69	19 (12)	
70–79	25 (16)	
80–89	8 (5)	
90–99	2 (1)	
Median age, y (IQR)	53 (37–65)	
Hospitalization, n = 128		
Yes	85 (66)	
No	43 (34)	
Death, n = 152		
Yes	5 (3)	
No	148 (97)	
Laboratory method, n = 152†		
Direct pathogen detection	66 (43)	
Antigen detection	5 (3)	
Bacterial culture	31 (20)	
Nucleic acid detection	33 (22)	
Indirect pathogen detection		
Antibody detection	102 (67)	
Forms of tularemia, n = 152‡		
Glandular	53 (35)	
Ulceroglandular	35 (23)	
Pneumonic	26 (17)	
Typhoidal	18 (12)	
Oropharyngeal	14 (9)	
Oculoglandular	5 (3)	
Unknown§	1	

*Values are no. (%) except as indicated. †Multiple tests possible per case. ‡On the basis of symptoms and exposure reported by case-patients. §One case had no symptoms available; hence, the form of tularemia could not be identified.

Of 142 cases for which information on animal exposure was available, 68% (n = 96) had known exposure, primarily to arthropod bites (45%, n = 43), including 28 tick bites. More than half of arthropod-borne infections occurred during 2019–2022 (n = 25). Among those 142 cases, <15% of cases reported contact with wild animals and 35% reported contact with domestic animals. Information on the consumption of contaminated food was available for just 1 case. Four case-patients reported the slaughter of wild animals, but no specific exposure details were available. Of 130 cases with professional exposure information, 31% had potential professional exposure, mainly from hunting and forestry work (43%, n = 17) or farm work (28%, n = 11). Of 84 cases that had other exposure information, 39% (n = 33) reported exposure to potentially contaminated environmental sources, including soil, mud and water sources.

**Figure 1:**
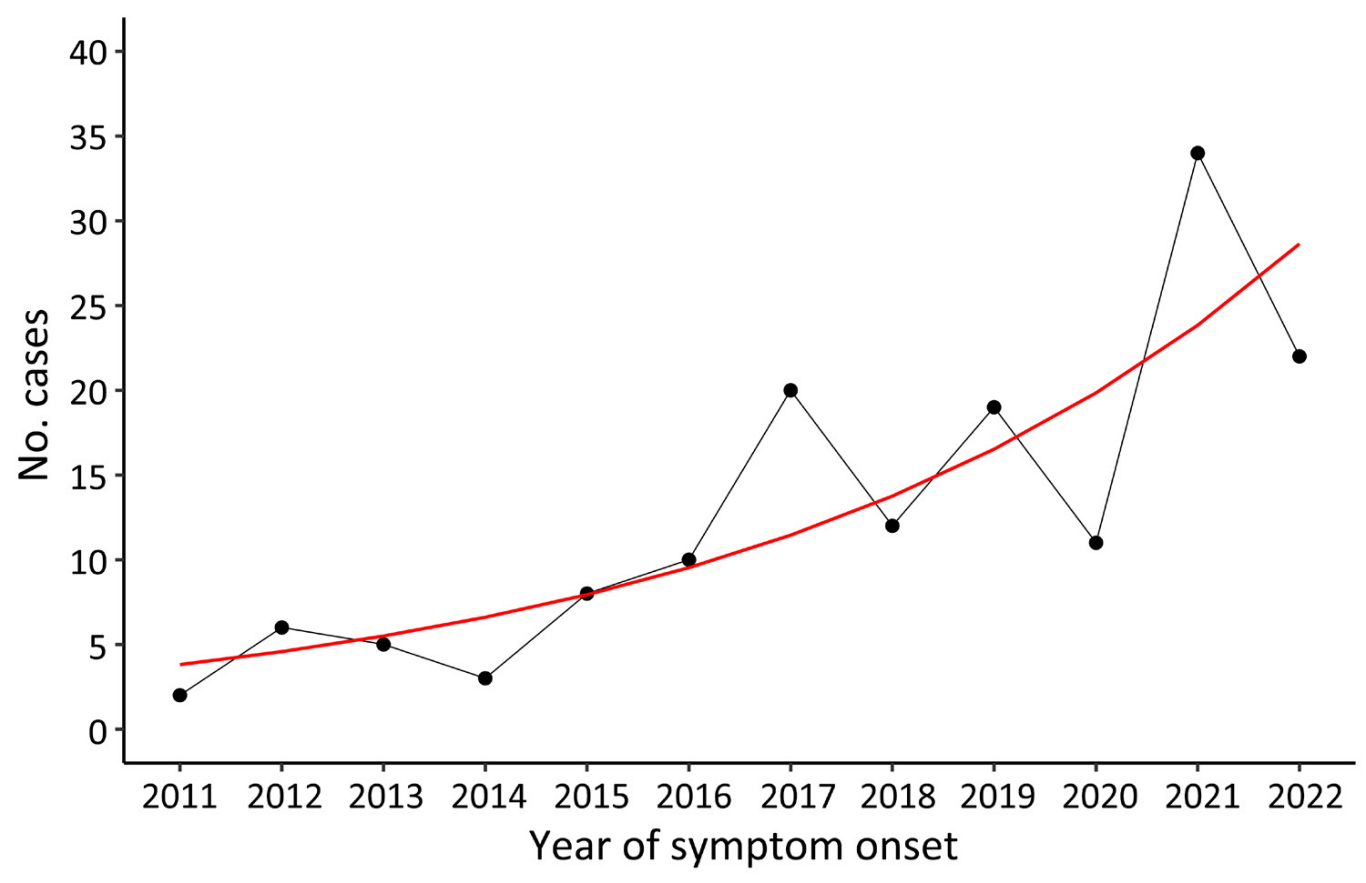
Notified human tularemia cases by year of symptom onset in study of epidemiology of tularemia among humans and animals, Baden-Wuerttemberg, Germany, 2012–2022. Red line indicates Poisson trend. Two cases had symptom onset in 2011 but were notified in 2012.

**Figure 2:**
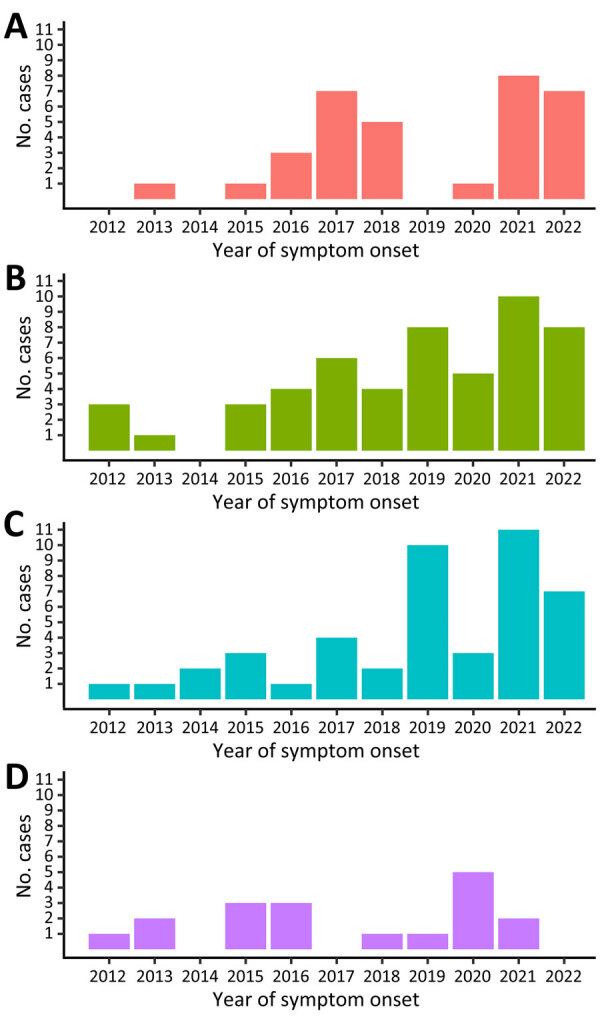
Seasonal distribution of notified human tularemia cases by year of symptom onset in study of epidemiology of tularemia among humans and animals, Baden-Wuerttemberg, Germany, 2012–2022. Notified cases are shown for spring (A), summer (B), autumn (C), and winter (D). Four cases with symptom onset in 2011 were excluded.

Poisson regression (Figure 1) showed a significant annual increase in tularemia cases by 20% (1.20, 95% CI 1.14–1.27; p<0.001). Periodicity could not be identified in data. Seasonal distribution of cases varied (Figure 2); the lowest case numbers were reported in winter. During 2021 and 2022, when case numbers were higher, cases were almost equally distributed across all seasons except winter.

### Epidemiology of Notified Animal Cases

**Figure 3:**
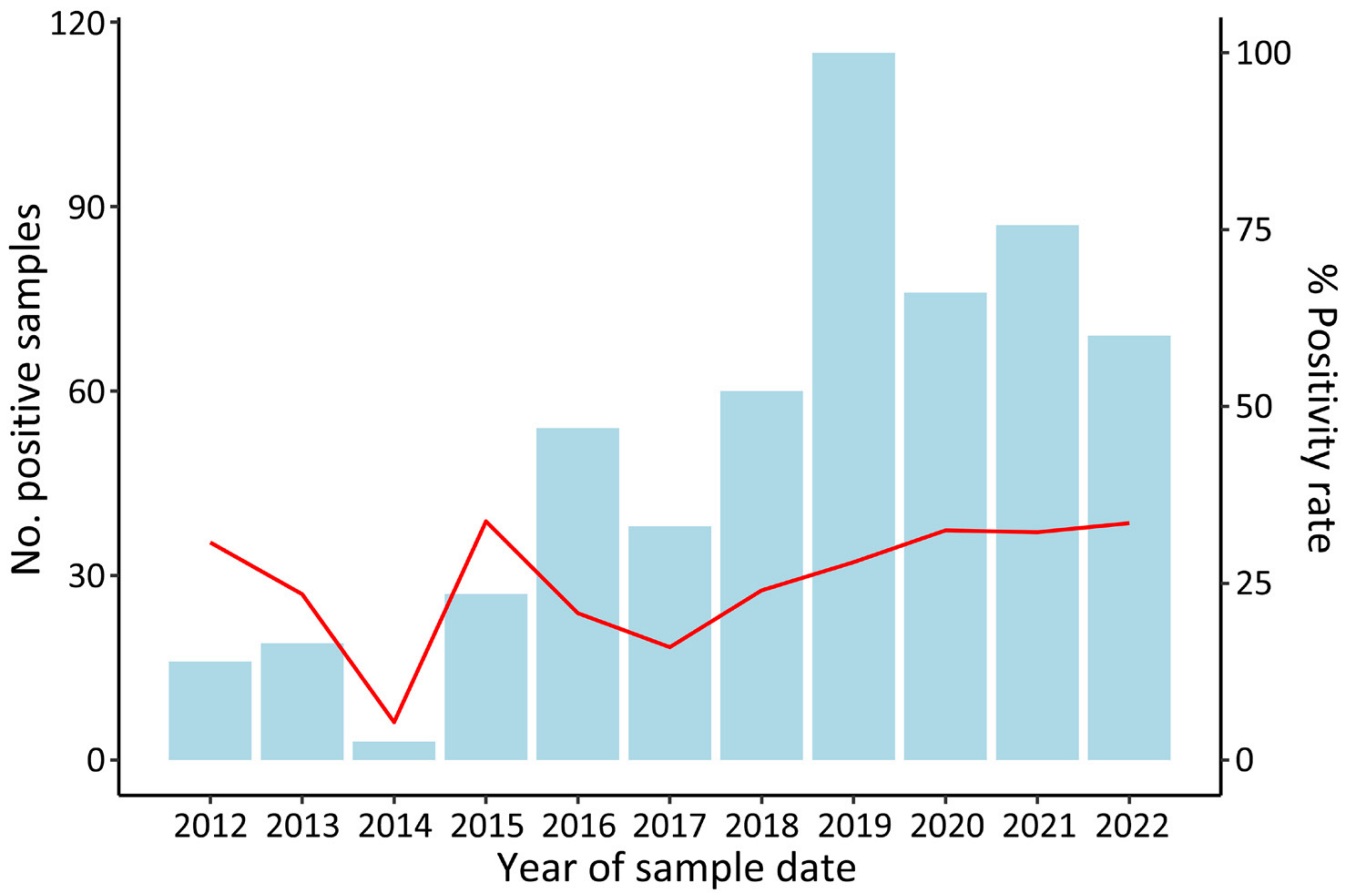
Number of positive animal samples by year of sample date and positivity rates in study of epidemiology of tularemia among humans and animals, Baden-Wuerttemberg, Germany, 2012–2022. Red line indicates positivity rates.

During 2012–2022, the Veterinary Analysis Agencies tested 2,145 animal samples for tularemia. Most samples were from brown hares (86%, n = 1,839), whereas other samples were collected from various animals, including wild boar, rodents, and domestic animals. Laboratory results were available for 2,138 samples, of which 564 (26%) tested positive for *F. tularensis.* Of those, 551 (98%) tested samples were from brown hares, with the rest from unspecified ground game (n = 7), wild birds (n = 4), 1 zoo animal, and 1 wild boar. Positivity rates fluctuated from 5% in 2012 to 34% in 2016 (Figure 3) but have steadily increased from 16% to 33% since 2017, alongside an increase in the number of samples tested.

### Geographic Distribution of Human and Animal Cases

**Figure 4:**
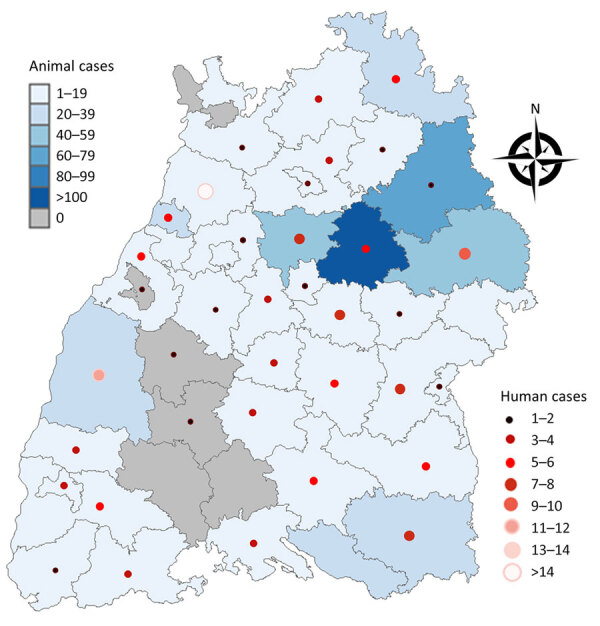
Geographic distribution of human and animal tularemia cases in study of epidemiology of tularemia among humans and animals, Baden-Wuerttemberg, Germany, 2012–2022.

Of the 44 districts in Baden-Wuerttemberg, 37 reported human or animal tularemia cases during 2012–2022 (Figure 4). Most human cases were reported from the districts of Karlsruhe (n = 15) and Ortenaukreis (n = 11). The districts Boeblingen, Enzkreis, Konstanz, and Rhein-Neckar-Kreis reported cases for the first time in 2021, and the city district of Heilbronn reported the first case in 2022.

Most animal cases were reported in the districts Rems-Murr-Kreis (n = 101), Schwaebisch Hall (n = 63), Ostalbkreis (n = 47), and Ludwigsburg (n = 41). In the other districts, the number of laboratory-confirmed animal cases ranged from 1 to 28 cases. Three of the districts reported human cases and no animal cases. The other districts reported animal cases but no human cases.

### Genomic Surveillance of *F. tularensis* subsp. *holarctica* in Baden-Wuerttemberg

We analyzed genome sequences of 115 *F. tularensis* subsp. *holarctica*, including 47 strains sequenced in this study and publicly available genome data of 68 strains (Appendix Table 1). All sequencing data produced in this study were added to the European Nucleotide Archive (Bioproject PRJEB40963). Twenty-four strains were isolated from human cases, whereas animal-derived strains were mainly isolated from brown hares (*Lepus europaeus*, n = 90); 1 strain was isolated from a Eurasian blue tit (*Cyanistes caeruleus*).

A minimum of 30-fold coverage was reached for all strains (Appendix Table 2). On average, 99.4% of raw reads belonged to the genus *Francisella* and 97.6% to *F. tularensis* subsp. *holarctica.* The chromosomal marker RD-1 for *F. tularensis* was detected in all genomes with an amplicon size of 924 bp, corresponding to *F. tularensis* subsp. *holarctica*. The mean assembly size was 1.79 Mbp, and the average N50 of the 115 assembled genomes was 42,988 bp (range 13,573–100,618 bp). The mean GC content was 32.2% with little variation.

**Table 2:** Summary of genotyping results in study of epidemiology of tularemia among humans and animals, Baden-Wuerttemberg, Germany, 2012–2022

Clade	Human	Animal
Major clades	B.6 (n = 21), B.12 (n = 3)	B.6 (n = 83), B.12 (n = 8)
#Subclades CanSNPer1	6	14
#Subclades CanSNPer2	14	29
#Cluster 5 cgSNPs	6	15

Canonical SNP-typing uses predefined lists of SNPs to assign major clades and subclades to each strain following a predefined decision tree. Two major clades were detected among the studied strains; most strains belonged to the major clade B.6 (n = 104) for both human isolates and animal-derived strains (Table 2; Appendix Table 1). Using CanSNPer1, we detected 14 canonical subclades. Although 6 subclades (B.33, B.34, B.45, B.49, B.61, and B.63) were shared between human and animal strains, 8 additional subclades were found only in animals. CanSNPer2 further subbranched many clades detected by CanSNPer1into 34 subclades. Within the dataset, 5 subclades (B.182, B.259, B.287, B.305, and B.91) were found only in human-derived strains, 20 were found only in animals, and 9 subclades were detected among strains from humans and animals. The strain from a Eurasian blue tit was isolated from a dead bird in the city of Freiburg im Breisgau. The sample was identified as *F. tularensis* subsp. *holarctica* by PCR and matrix-assisted laser desorption/ionization time-of-flight mass spectrometry and was typed as clade B.6, subclade B.49, which was also detected in several strains isolated from hares and humans in Baden-Wuerttemberg.

**Figure 5:**
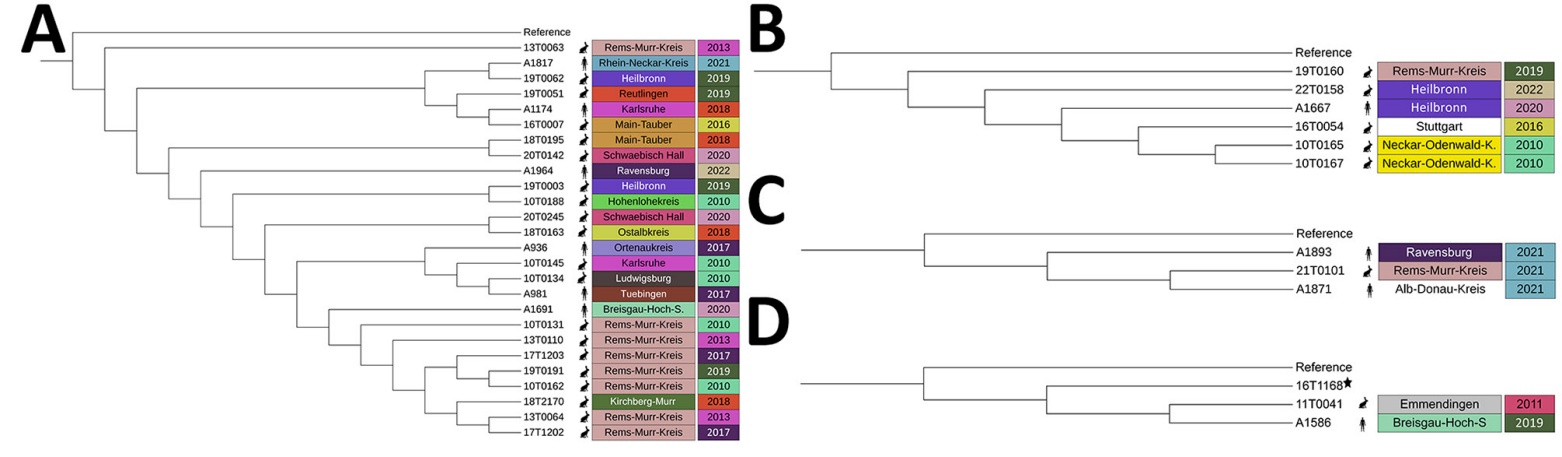
Phylogenetic trees of clusters containing *Francisella* strains isolated from humans and animals in study of epidemiology of tularemia among humans and animals, Baden-Wuerttemberg, Germany, 2012–2022. Colors indicate host, county, and year of isolation. Strain 16T1186 is not a member of cluster 10 and was added only to reach minimal amount of strains for phylogeny.

The variable core-genome alignment contains all genomic positions of the reference genome, which exist in all of the strains and are variable in >1 strain. This alignment consisted of 24,359 bp and enabled the construction of a phylogenetic tree (Appendix Figure). The phylogeny of *F. tularensis* in Baden-Wuerttemberg constitutes 2 major branches in accordance with the major canonical clades B.6 and B.12, whereas clusters of genetically similar strains mostly follow the canonical subclades. Strains isolated from patients are closely related to strains obtained from animals, because human-derived strains do not form outgroups and they cluster with animal strains. The pairwise number of cgSNPs between strains ranged from 0 to 23,595 cgSNPs with a median of 8 cgSNPs (Appendix Table 1). Hierarchical clustering was performed to group strains into genomically highly similar clusters. Using a cutoff of 5 cgSNPs (*1*), 83/115 (72%) strains were clustered into 15 groups (Appendix Table 1). Although 11 clusters contained strains that were exclusively isolated from animals, 4 clusters (clusters 1, 3, 8, and 10) contained strains from humans and animals (Figure 5; Appendix Table 1). The largest cluster (cluster 1) contained 26 strains (Figure 5, panel A), of which 5 were isolated from patients during 2017–2022 at different locations in Baden-Wuerttemberg. For example, the *F. tularensis* strain A1174 isolated from a patient in the city of Karlsruhe was 1 cgSNP different from 4 isolates from hares in southern Baden-Wuerttemberg (Appendix Table 1). Among those 6 isolates, 4 case-patients had been exposed to arthropod bites (3 tick bites and 2 mosquito bites). One of the case-patients had no known risk exposure but reported owning a cat. One case was in a farm worker who was reportedly exposed to sewage through work. Cluster 3 contained 6 strains, including the human isolate A-1667 from 2020 in Heilbronn and the hare-derived strain 22T0158 from the same city (Figure 5, panel B). The strain 16T0054 was isolated from a hare in Stuttgart and 1 cgSNP different from A-1667. No exposure risks were reported in that case, and the mode of transmission remained unclear. Two strains from patients and 1 strain from a hare, all isolated in 2021, grouped within cluster 8 (Figure 5, panel C). Strain A1871 was isolated from a case-patient without exposure risk at work but who was active in a private garden and, therefore, potentially had contact with rodent feces. Its genome was 1 cgSNP different from the hare-derived strain 21T0101 (Appendix Table 1). Strain A1893 was isolated from a farmer who had contact with mice and feces from rodents. Cluster 10 contained 1 human and 1 animal-derived strain. The case-patient reported contact with a dog but no other risk factors (Figure 5, panel D).

In general, canonical (sub)clades of CanSNPer1 are either identical when using CanSNPer2 or split into several subclades (Appendix Table 1). Using CanSNPer1, we found 51/115 strains (44%) belong to subclade B.45. Of those 51 strains, 15 belong to B.45 when using CanSNPer2, whereas 36 belong to 10 different subclades. Subclades are comparable to hierarchical clusters. For example, hierarchical clusters 3, 4, 5, 7, and 11 are completely identical to respective subclades identified by CanSNPer2. Cluster 1 is split into 5 subclades, whereas subclade B.63 is split into hierarchical clusters 6 and 8.

## Discussion

Baden-Wuerttemberg is among the states in Germany with the highest human incidence rates of tularemia (*5*,*6*); cases peaked in 2021. Despite decreased numbers in 2022, they remain above the 2012–2020 average. Animal surveillance data shows an increase in positivity rates from 16% to 33% during 2017–2022. A human seroprevalence study in Baden-Wuerttemberg found a 2.34% infection rate (*27*), suggesting many cases remain undetected and underreported. Besides that, tularemia in humans manifests in various symptoms and might rarely be considered as a differential diagnosis for febrile diseases and diseases accompanied by enlarged lymph nodes (*4*,*5*), which can lead to late diagnosis. The median time from symptom onset to reporting was 29 days, despite the incubation period of 3–5 days (*8*,*9*). Twelve percent of cases were reported with a lag time of >96 days after symptom onset. Those findings suggest delayed diagnosis rather than delayed reporting, which could result in inadequate patient treatment (*28*).

The 20% annual increase in human tularemia cases might reflect either increasing incidence of the disease or improved detection and reporting. The increase in pneumonic tularemia could be linked to a greater focus on pneumonia diagnosis because of COVID-19. More awareness among healthcare professionals and clinicians might have also contributed to increased reporting of human tularemia cases.

Although no seasonal pattern was identified in human surveillance data, case numbers were lowest in winter. Climate change might contribute to increasing tularemia cases, because research shows the disease is sensitive to small temperature changes (*29*). Predictions in Austria (*30*) about the spread of tularemia because of climate change proved accurate; new cases appeared in previously unaffected areas (*31*). Baden-Wuerttemberg will also experience rising temperatures (*32*,*33*), potentially affecting arthropodborne transmission. Among the 96 human cases who had animal exposure, 45% of infections were attributed to arthropod bites; >50% were reported in the 4 previous years. That increase might reflect longer periods of arthropod activity or increased abundances because of warmer temperatures (*33*–*35*) or a change of leisure behavior (*5*). The peak of human cases in 2021 might be explained by an increase in outdoor activities during COVID-19 lockdowns (*36*,*37*).

WGS-based genotyping of *F. tularensis* subsp. *holarctica* in Baden-Wuerttemberg displayed the typical phylogeography of this pathogen in Germany (*15*,*16*): most strains in the southwest belong to major clade B.6, strains belonging to specific subclades or high-resolution SNP clusters might spread between different regions, and subclades and SNP clusters are shared between animals (typically hares) and humans. Although this study detected some subclades and SNP clusters only in animals, this finding might be caused by a sampling-bias (i.e., genotypes might be detected in cases from other federal states or at later time points in Baden-Wurttemberg). To our knowledge, no host-specific genotypes of *F. tularensis* subsp. *holarctica* have been detected in Germany. In France, no association between (sub)clades and hosts was detected, whereas spatiotemporal clusters existed in Germany (*38*). Although the pathogen has been detected in birds before we sequenced *F. tularensis* subsp. *holarctica* from a Eurasian blue tit was sequenced, which suggests the possibility of avian spread. 

WGS data were available only for some of the human and animal cases. Sampling and cultivation are not always possible, and WGS of *F. tularensis* is not established in many laboratories in the human and animal reporting system in Germany. Small geographic regions are often inhabited by isolates of several subclades, and genomically near-identical isolates of the same subclades might disperse across larger distances (*15*). However, even high-resolution genotyping is not always conclusive regarding source attribution. This problem is further complicated because diverse *F. tularensis* clones might cause human outbreaks (*39*). In cases in which epidemiologic analyses identified several potential sources of infection, genotyping might be useful to exclude strains with larger genomic distances. Detailed epidemiology information of animal and human strains is needed to understand genomic links. As previously shown (*15*), cgSNP typing followed by hierarchical clustering is highly comparable to canonical subclades. CanSNPer2 introduced many new subclades further subbranching subclades often found in Germany (e.g., B.45, B.49), which improved resolution over CanSNPer1. Because canonical subclades rely on SNP differences, hierarchical clusters using 5 cgSNPs as the threshold might split into several canonical subclades (e.g., hierarchical cluster 1). Using a threshold of 0 cgSNP for clustering circumvents this situation but clusters fewer strains.

Animal surveillance data shows most positive samples originated from brown hares, but their population density in Baden-Wuerttemberg (10.3–15.6/km^2^) did not increase unusually (*40*). Despite brown hares being regarded as indicator animals, the increase in positivity rates among hares is not reflected in human surveillance data. Therefore, considering other natural animal and environmental reservoirs is crucial (*11*,*41*,*42*). Seropositivity in other indicator animals, such as wild boars and foxes, which live in larger territories than hares, might help to identify larger tularemia-endemic regions (*43*).

We found no geographic link between human and animal tularemia cases. Some districts reported human cases without animal cases, and some reported the contrary. Human surveillance data often lacks specific exposure locations; many cases are assumed to be from the notifying district. Travel or hunting in other areas could be source of infection. Foodborne exposures were rarely reported, and <15% of cases involved wild animal contact, even though many case-patients were hunters and had butchered animals. Better collaborations with local health authorities are needed to improve surveillance data and understand geographic disease spread and risk factors.

In conclusion, the substantial annual increase in tularemia cases in Baden-Wuerttemberg could reflect either increased disease prevalence or improved detection and reporting. To clarify this trend, examining factors such as environmental changes, public health interventions, and diagnostic practices will be critical. Routine genome sequencing of human and animal isolates should be combined with detailed epidemiologic tracing to improve knowledge of potential infection sources.

Appendix Additional information about epidemiology of tularemia among humans and animals, Baden-Wuerttemberg, Germany, 2012–2022.
