# Supplementary material for: Epidemiology of Tularemia among Humans and Animals, Baden-Wuerttemberg, Germany, 2012–2022
Source: Emerg Infect Dis. 2025 Apr;31(4):678–88. doi: 10.3201/eid3104.240414 (PMC11950275; doi:10.3201/eid3104.240414)
Supplement: Appendix — Additional information about epidemiology of tularemia among humans and animals, Baden-Wuerttemberg, Germany, 2012–2022. [file 24-0414-Techapp-s1.pdf]

Article DOI: https://doi.org/10.3201/eid3104.240414

*EID cannot ensure accessibility for supplementary materials supplied by authors. Readers who have difficulty accessing supplementary content should contact the authors for assistance.*

# Epidemiology of Tularemia among Humans and Animals, Baden-Wuerttemberg, Germany, 2012–2022

## Appendix

**Appendix Table 1.** Epidemiologic information and genotyping results of 115 analyzed strains of *F. tularensis* subsp. *holarctica* from Baden-Wuerttemberg.

| ID | Strain Name | Pubmed. BioProject. Accession | Year | Institute* | City | County (district) | Host | Major Clade | SubCan Snper1 | SubCan Snper2 | SNP_Path | Max SNPs_1 | Max SNPs_5 |
|---|---|---|---|---|---|---|---|---|---|---|---|---|---|
| 08T0014 | 08T0014 | 33291395 | 2008 | FLI | Flacht-Wiessach | Boeblingen | Lepus europaeus | B.6 | B.63 | B.63 | T/N.1;T.1;B.1;B.2;B.3;B.5;B.6;B.10;B.11;B.44;B.228;B.46;B.47;B.63 | 6 | 6 |
| 08T0015 | 08T0015 | 33291395 | 2008 | FLI | Hoefingen | Boeblingen | Lepus europaeus | B.6 | B.7 | B.138 | T/N.1;B.217;B.74;B.45;B.166;B.138 | | |
| 09T0109 | 09T0109 | 33291395 | 2009 | FLI | Markdorf | Friedrichshafen | Lepus europaeus | B.6 | B.60 | B.60 | T/N.1;T.1;B.1;B.2;B.3;B.5;B.6;B.10;B.11;B.44;B.45;B.50;B.60 | | 9 |
| 09T0146 | 09T0146 | 33291395 | 2009 | FLI | Zeilwald | Ludwigsburg | Lepus europaeus | B.6 | B.61 | B.281 | T/N.1;T.1;B.1;B.2;B.3;B.5;B.6;B.10;B.11;B.44;B.45;B.50;B.61;B.281 | 1 | 2 |
| 10T0131 | 10T0131 | 33291395 | 2010 | FLI | Oppenweiler | Rems-Murr-Kreis | Lepus europaeus | B.6 | B.45 | B.87 | T/N.1;T.1;B.1;B.2;B.3;B.5;B.6;B.10;B.11;B.44;B.45;B.87 | 2 | 1 |
| 10T0134 | 10T0134 | 33291395 | 2010 | FLI | Ludwigsburg | Ludwigsburg | Lepus europaeus | B.6 | B.45 | B.45 | T/N.1;T.1;B.1;B.2;B.3;B.5;B.6;B.10;B.11;B.44;B.45 | 3 | 1 |
| 10T0145 | 10T0145 | 33291395 | 2010 | FLI | Oestringen | Karlsruhe | Lepus europaeus | B.6 | B.45 | B.45 | T/N.1;T.1;B.1;B.2;B.3;B.5;B.6;B.10;B.11;B.44;B.45 | 3 | 1 |
| 10T0162 | 10T0162 | 33291395 | 2010 | FLI | Oppenweiler | Rems-Murr-Kreis | Lepus europaeus | B.6 | B.45 | B.87 | T/N.1;T.1;B.1;B.2;B.3;B.5;B.6;B.10;B.11;B.44;B.45;B.87 | 2 | 1 |
| 10T0165 | 10T0165 | 33291395 | 2010 | FLI | Buchen | Neckar-Odenwald-Kreis | Lepus europaeus | B.6 | B.45 | B.87 | T/N.1;T.1;B.1;B.2;B.3;B.5;B.6;B.10;B.11;B.44;B.45;B.87 | 9 | 3 |
| 10T0167 | 10T0167 | 33291395 | 2010 | FLI | Heidelberg | Neckar-Odenwald-Kreis | Lepus europaeus | B.6 | B.45 | B.87 | T/N.1;T.1;B.1;B.2;B.3;B.5;B.6;B.10;B.11;B.44;B.45;B.87 | 9 | 3 |
| 10T0168 | 10T0168 | 33291395 | 2010 | FLI | Duttenberg | Heilbronn | Lepus europaeus | B.6 | B.45 | B.45 | T/N.1;T.1;B.1;B.2;B.3;B.5;B.6;B.10;B.11;B.44;B.45 | | |
| 10T0188 | 10T0188 | 33291395 | 2010 | FLI | Mulfingen | Hohenlohekreis | Lepus europaeus | B.6 | B.45 | B.45 | T/N.1;T.1;B.1;B.2;B.3;B.5;B.6;B.10;B.11;B.44;B.45 | 10 | 1 |
| 10T0189 | 10T0189 | 33291395 | 2010 | FLI | Schwanau | Ortenaukreis | Lepus europaeus | B.6 | B.49 | B.49 | T/N.1;T.1;B.1;B.2;B.3;B.5;B.6;B.10;B.11;B.44;B.45;B.49 | | |

| ID | Strain Name | Pubmed. BioProject. Accession | Year | Institute* | City | County (district) | Host | Major Clade | SubCan Snper1 | SubCan Snper2 | SNP_Path | Max SNPs_1 | Max SNPs_5 |
|---|---|---|---|---|---|---|---|---|---|---|---|---|---|
| 10T0195 | 10T0195 | 33291395 | 2010 | FLI | Loerrach | Loerrach | Lepus europaeus | B.6 | B.53 | B.53 | T/N.1;T.1;B.1;B.2;B.3;B.5;B.6;B.10;B.11;B.44;B.45;B.53 | | |
| 11T0041 | 11T0041 | 33291395 | 2011 | FLI | Teningen | Emmendingen | Lepus europaeus | B.6 | B.49 | B.49 | T/N.1;T.1;B.1;B.2;B.3;B.5;B.6;B.10;B.11;B.44;B.45;B.49 | | 10 |
| 12T0023 | 12T0023 | 33291395 | 2012 | FLI | Hochdorf | Ludwigsburg | Lepus europaeus | B.6 | B.61 | B.281 | T/N.1;T.1;B.1;B.2;B.3;B.5;B.6;B.10;B.11;B.44;B.45;B.50;B.61;B.281 | 1 | 2 |
| 12T0048 | 12T0048 | 33291395 | 2012 | FLI | Eberdingen | Ludwigsburg | Lepus europaeus | B.6 | B.61 | B.281 | T/N.1;T.1;B.1;B.2;B.3;B.5;B.6;B.10;B.11;B.44;B.45;B.50;B.61;B.281 | | 2 |
| 13T0036 | 13T0036 | 33291395 | 2013 | FLI | Aspach | Ludwigsburg | Lepus europaeus | B.6 | B.61 | B.281 | T/N.1;T.1;B.1;B.2;B.3;B.5;B.6;B.10;B.11;B.44;B.45;B.50;B.61;B.281 | 1 | 2 |
| 13T0063 | 13T0063 | 33291395 | 2013 | FLI | Kernen | Rems-Murr-Kreis | Lepus europaeus | B.6 | B.45 | B.45 | T/N.1;T.1;B.1;B.2;B.3;B.5;B.6;B.10;B.11;B.44;B.45 | 3 | 1 |
| 13T0064 | 13T0064 | 33291395 | 2013 | FLI | Aspach | Rems-Murr-Kreis | Lepus europaeus | B.6 | B.45 | B.87 | T/N.1;T.1;B.1;B.2;B.3;B.5;B.6;B.10;B.11;B.44;B.45;B.87 | 2 | 1 |
| 13T0110 | 13T0110 | 33291395 | 2013 | FLI | Oppenweiler | Rems-Murr-Kreis | Lepus europaeus | B.6 | B.45 | B.87 | T/N.1;T.1;B.1;B.2;B.3;B.5;B.6;B.10;B.11;B.44;B.45;B.87 | 2 | 1 |
| 14T0026 | 14T0026 | 33291395 | 2014 | FLI | Oberriexingen | Ludwigsburg | Lepus europaeus | B.6 | B.61 | B.281 | T/N.1;T.1;B.1;B.2;B.3;B.5;B.6;B.10;B.11;B.44;B.45;B.50;B.61;B.281 | 1 | 2 |
| 14T0098 | 14T0098 | 33291395 | 2014 | FLI | Bad Saeckingen | Waldshut | Homo sapiens | B.6 | B.61 | B.279 | T/N.1;T.1;B.1;B.2;B.3;B.5;B.6;B.10;B.11;B.44;B.45;B.50;B.61;B.279 | | |
| 15T0224 | 15T0224 | 33291395 | 2015 | FLI | Oppenweiler | Rems-Murr-Kreis | Lepus europaeus | B.6 | B.61 | B.281 | T/N.1;T.1;B.1;B.2;B.3;B.5;B.6;B.10;B.11;B.44;B.45;B.50;B.61;B.281 | 1 | 2 |
| 15T0767 | 15T0767 | 33291395 | 2015 | FLI | Ludwigsburg | Ludwigsburg | Lepus europaeus | B.6 | B.61 | B.281 | T/N.1;T.1;B.1;B.2;B.3;B.5;B.6;B.10;B.11;B.44;B.45;B.50;B.61;B.281 | 1 | 2 |
| 15T0808 | 15T0808 | 33291395 | 2015 | FLI | Enz-Kreis | Enz-Kreis | Lepus europaeus | B.6 | B.45 | B.302 | T/N.1;T.1;B.1;B.2;B.3;B.5;B.6;B.10;B.11;B.44;B.45;B.87;B.302 | | 11 |
| 16T0007 | 16T0007 | 33291395 | 2016 | FLI | Dertingen | Main-Tauber | Lepus europaeus | B.6 | B.45 | B.45 | T/N.1;T.1;B.1;B.2;B.3;B.5;B.6;B.10;B.11;B.44;B.45 | 7 | 1 |
| 16T0022 | 16T0022 | 33291395 | 2016 | FLI | Stuttgart | Stuttgart | Lepus europaeus | B.6 | B.45 | B.45 | T/N.1;T.1;B.1;B.2;B.3;B.5;B.6;B.10;B.11;B.44;B.45 | 11 | 12 |
| 16T0051 | 16T0051 | 33291395 | 2016 | FLI | Stuttgart | Stuttgart | Lepus europaeus | B.6 | B.61 | B.281 | T/N.1;T.1;B.1;B.2;B.3;B.5;B.6;B.10;B.11;B.44;B.45;B.50;B.61;B.281 | | 2 |
| 16T0052 | 16T0052 | 33291395 | 2016 | FLI | Stuttgart | Stuttgart | Lepus europaeus | B.6 | B.61 | B.279 | T/N.1;T.1;B.1;B.2;B.3;B.5;B.6;B.10;B.11;B.44;B.45;B.50;B.61;B.279 | 12 | 2 |
| 16T0053 | 16T0053 | 33291395 | 2016 | FLI | Stuttgart | Stuttgart | Lepus europaeus | B.6 | B.61 | B.279 | T/N.1;T.1;B.1;B.2;B.3;B.5;B.6;B.10;B.11;B.44;B.45;B.50;B.61;B.279 | 12 | 2 |
| 16T0054 | 16T0054 | 33291395 | 2016 | FLI | Stuttgart | Stuttgart | Lepus europaeus | B.6 | B.45 | B.87 | T/N.1;T.1;B.1;B.2;B.3;B.5;B.6;B.10;B.11;B.44;B.45;B.87 | 13 | 3 |
| 16T0621 | 16T0621 | 33291395 | 2016 | FLI | Ludwigsburg | Ludwigsburg | Lepus europaeus | B.6 | B.45 | B.45 | T/N.1;T.1;B.1;B.2;B.3;B.5;B.6;B.10;B.11;B.44;B.45 | 11 | 12 |
| 16T0622 | 16T0622 | 33291395 | 2016 | FLI | Schwaebisch-Hall | Schwaebisch Hall | Lepus europaeus | B.6 | B.62 | B.62 | T/N.1;T.1;B.1;B.2;B.3;B.5;B.6;B.10;B.11;B.44;B.45;B.51;B.62 | | 13 |
| 16T0623 | 16T0623 | 33291395 | 2016 | FLI | Eltershofen | Schwaebisch Hall | Lepus europaeus | B.6 | B.45 | B.255 | T/N.1;T.1;B.1;B.2;B.3;B.5;B.6;B.10;B.11;B.44;B.45;B.251;B.101;B.252;B.255 | | |
| 16T1168 | 16T1168 | 33291395 | 2016 | FLI | Goeppingen | Goeppingen | Lepus europaeus | B.12 | B.39 | B.215 | T/N.1;T.1;B.1;B.2;B.3;B.5;B.12;B.72;B.39;B.215 | 14 | 14 |

| ID | Strain Name | Pubmed. BioProject. Accession | Year | Institute* | City | County (district) | Host | Major Clade | SubCan Snper1 | SubCan Snper2 | SNP_Path | Max SNPs_ 1 | Max SNPs_ 5 |
|---|---|---|---|---|---|---|---|---|---|---|---|---|---|
| 16T1169 | 16T1169 | 33291395 | 2016 | FLI | Goeppingen | Goeppingen | Lepus europaeus | B.12 | B.39 | B.215 | T/N.1;T.1;B.1;B.2;B.3;B.5;B.12;B.72;B.39;B.215 | 14 | 14 |
| 16T1176 | 16T1176 | 33291395 | 2016 | FLI | Weckrieden | Schwaebisch Hall | Lepus europaeus | B.12 | B.33 | B.103 | T/N.1;T.1;B.1;B.2;B.3;B.5;B.12;B.72;B.13;B.26;B.42;B.168;B.66; B.33;B.103 | 4 | 4 |
| 17T1202 | 17T1202 | 33291395 | 2017 | FLI | Kirchberg-Murr | Rems-Murr-Kreis | Lepus europaeus | B.6 | B.45 | B.87 | T/N.1;T.1;B.1;B.2;B.3;B.5;B.6;B.10;B.11;B.44;B.45;B.87 | 2 | 1 |
| 17T1203 | 17T1203 | 33291395 | 2017 | FLI | Kirchberg-Murr | Rems-Murr-Kreis | Lepus europaeus | B.6 | B.45 | B.87 | T/N.1;T.1;B.1;B.2;B.3;B.5;B.6;B.10;B.11;B.44;B.45;B.87 | 2 | 1 |
| 17T1415 | 17T1415 | 33291395 | 2017 | FLI | Wasseralfingen Spitzwald | Aalen | Lepus europaeus | B.6 | B.45 | B.240 | T/N.1;T.1;B.1;B.2;B.3;B.5;B.6;B.10;B.11;B.44;B.45;B.240 | 5 | 5 |
| 17T1542 | 17T1542 | 33291395 | 2017 | FLI | Backnag | Rems-Murr-Kreis | Lepus europaeus | B.6 | B.61 | B.281 | T/N.1;T.1;B.1;B.2;B.3;B.5;B.6;B.10;B.11;B.44;B.45;B.50;B.61;B. 281 | 1 | 2 |
| 18T0029 | 18T0029 | 33291395 | 2018 | FLI | Obergroenningen | Obergroenningen | Lepus europaeus | B.6 | B.45 | B.92 | T/N.1;T.1;B.1;B.2;B.3;B.5;B.6;B.10;B.11;B.44;B.45;B.92 | | |
| 18T0158 | 18T0158 | 33291395 | 2018 | FLI | Abtsgmuend | Ostalbkreis | Lepus europaeus | B.12 | B.33 | B.103 | T/N.1;T.1;B.1;B.2;B.3;B.5;B.12;B.72;B.13;B.26;B.42;B.168;B.66; B.33;B.103 | 4 | 4 |
| 18T0161 | 18T0161 | 33291395 | 2018 | FLI | Obersulm | Heilbronn | Lepus europaeus | B.6 | B.61 | B.281 | T/N.1;T.1;B.1;B.2;B.3;B.5;B.6;B.10;B.11;B.44;B.45;B.50;B.61;B. 281 | 1 | 2 |
| 18T0163 | 18T0163 | 33291395 | 2018 | FLI | Aalen | Ostalbkreis | Lepus europaeus | B.6 | B.45 | B.45 | T/N.1;T.1;B.1;B.2;B.3;B.5;B.6;B.10;B.11;B.44;B.45 | | 1 |
| 18T0164 | 18T0164 | 33291395 | 2018 | FLI | Althengstett | Calw | Lepus europaeus | B.6 | B.61 | B.281 | T/N.1;T.1;B.1;B.2;B.3;B.5;B.6;B.10;B.11;B.44;B.45;B.50;B.61;B. 281 | | 2 |
| 18T0195 | 18T0195 | 33291395 | 2018 | FLI | Bad Mergentheim-Loeffelstein | Main-Tauber | Lepus europaeus | B.6 | B.49 | B.266 | T/N.1;T.1;B.1;B.2;B.3;B.5;B.6;B.10;B.11;B.44;B.45;B.49;B.266 | 15 | 1 |
| 18T0198 | 18T0198 | 33291395 | 2018 | FLI | Igersheim | Main-Tauber | Lepus europaeus | B.6 | B.45 | B.248 | T/N.1;T.1;B.1;B.2;B.3;B.5;B.6;B.10;B.11;B.44;B.45;B.247;B.248 | | |
| 18T2158 | 18T2158 | 33291395 | 2018 | FLI | Niederalfingen-Neuler | Ostalbkreis | Lepus europaeus | B.6 | B.45 | B.101 | T/N.1;T.1;B.1;B.2;B.3;B.5;B.6;B.10;B.11;B.44;B.45;B.251;B.101 | | |
| 18T2163 | 18T2163 | 33291395 | 2018 | FLI | Aalen | Ostalbkreis | Lepus europaeus | B.12 | B.34 | B.34 | T/N.1;T.1;B.1;B.2;B.3;B.5;B.12;B.72;B.13;B.26;B.42;B.168;B.66; B.33;B.34 | | |
| 18T2170 | 18T2170 | 33291395 | 2018 | FLI | Kirchberg-Murr | Kirchberg-Murr | Lepus europaeus | B.6 | B.45 | B.87 | T/N.1;T.1;B.1;B.2;B.3;B.5;B.6;B.10;B.11;B.44;B.45;B.87 | 2 | 1 |
| 19T0002 | 19T0002 | 33291395 | 2019 | FLI | Tuebingen | Tuebingen | Lepus europaeus | B.6 | B.61 | B.281 | T/N.1;T.1;B.1;B.2;B.3;B.5;B.6;B.10;B.11;B.44;B.45;B.50;B.61;B. 281 | 16 | 2 |
| 19T0003 | 19T0003 | 33291395 | 2019 | FLI | Wuestenrot | Heilbronn | Lepus europaeus | B.6 | B.45 | B.45 | T/N.1;T.1;B.1;B.2;B.3;B.5;B.6;B.10;B.11;B.44;B.45 | 10 | 1 |
| 19T0037 | 19T0037 | 33291395 | 2019 | FLI | Reutlingen | Reutlingen | Lepus europaeus | B.6 | B.45 | B.248 | T/N.1;T.1;B.1;B.2;B.3;B.5;B.6;B.10;B.11;B.44;B.45;B.247;B.248 | 17 | 15 |
| 19T0045 | 19T0045 | 33291395 | 2019 | FLI | Tuebingen | Tuebingen | Lepus europaeus | B.6 | B.61 | B.281 | T/N.1;T.1;B.1;B.2;B.3;B.5;B.6;B.10;B.11;B.44;B.45;B.50;B.61;B. 281 | | 2 |
| 19T0046 | 19T0046 | 33291395 | 2019 | FLI | Reutlingen | Reutlingen | Lepus europaeus | B.6 | B.45 | B.87 | T/N.1;T.1;B.1;B.2;B.3;B.5;B.6;B.10;B.11;B.44;B.45;B.87 | | |
| 19T0051 | 19T0051 | 33291395 | 2019 | FLI | Reutlingen | Reutlingen | Lepus europaeus | B.6 | B.45 | B.45 | T/N.1;T.1;B.1;B.2;B.3;B.5;B.6;B.10;B.11;B.44;B.45 | 3 | 1 |
| 19T0062 | 19T0062 | 33291395 | 2019 | FLI | Lauffen am Neckar | Heilbronn | Lepus europaeus | B.6 | B.45 | B.45 | T/N.1;T.1;B.1;B.2;B.3;B.5;B.6;B.10;B.11;B.44;B.45 | 7 | 1 |

| ID | Strain Name | Pubmed. BioProject. Accession | Year | Institute* | City | County (district) | Host | Major Clade | SubCan Snper1 | SubCan Snper2 | SNP_Path | Max SNPs_ 1 | Max SNPs_ 5 |
|---|---|---|---|---|---|---|---|---|---|---|---|---|---|
| 19T0063 | 19T0063 | 33291395 | 2019 | FLI | Tuebingen | Tuebingen | Lepus europaeus | B.6 | B.61 | B.281 | T/N.1;T.1;B.1;B.2;B.3;B.5;B.6;B.10;B.11;B.44;B.45;B.50;B.61;B.281 | 16 | 2 |
| 19T0098 | 19T0098 | 33291395 | 2019 | FLI | Oehringen | Hohenlohekreis | Lepus europaeus | B.6 | B.55 | B.55 | T/N.1;T.1;B.1;B.2;B.3;B.5;B.6;B.10;B.11;B.44;B.45;B.55 | | |
| 19T0118 | 19T0118 | | 2019 | FLI | Schwaebisch Hall | Schwaebisch Hall | Lepus europaeus | B.12 | B.33 | B.103 | T/N.1;T.1;B.1;B.2;B.3;B.5;B.12;B.72;B.13;B.26;B.42;B.168;B.66;B.33;B.103 | 4 | 4 |
| 19T0120 | 19T0120 | | 2019 | FLI | Schwaebisch Hall | Schwaebisch Hall | Lepus europaeus | B.12 | B.33 | B.103 | T/N.1;T.1;B.1;B.2;B.3;B.5;B.12;B.72;B.13;B.26;B.42;B.168;B.66;B.33;B.103 | 4 | 4 |
| 19T0160 | 19T0160 | | 2019 | FLI | Beutelsbach | Rems-Murr-Kreis | Lepus europaeus | B.6 | B.45 | B.87 | T/N.1;T.1;B.1;B.2;B.3;B.5;B.6;B.10;B.11;B.44;B.45;B.87 | | 3 |
| 19T0161 | 19T0161 | | 2019 | FLI | Wimsheim | Enz-Kreis | Lepus europaeus | B.6 | B.45 | B.302 | T/N.1;T.1;B.1;B.2;B.3;B.5;B.6;B.10;B.11;B.44;B.45;B.87;B.302 | | 11 |
| 19T0165 | 19T0165 | | 2019 | FLI | Grossbettlinge | Esslingen | Lepus europaeus | B.6 | B.45 | B.238 | T/N.1;T.1;B.1;B.2;B.3;B.5;B.6;B.10;B.11;B.44;B.45;B.238 | | |
| 19T0191 | 19T0191 | | 2019 | FLI | Kirchberg | Rems-Murr-Kreis | Lepus europaeus | B.6 | B.45 | B.87 | T/N.1;T.1;B.1;B.2;B.3;B.5;B.6;B.10;B.11;B.44;B.45;B.87 | 2 | 1 |
| 19T0194 | 19T0194 | | 2019 | FLI | | Rems-Murr-Kreis | Lepus europaeus | B.6 | B.45 | B.240 | T/N.1;T.1;B.1;B.2;B.3;B.5;B.6;B.10;B.11;B.44;B.45;B.240 | 5 | 5 |
| 19T0240 | 19T0240 | | 2019 | FLI | | Rems-Murr-Kreis | Lepus europaeus | B.6 | B.45 | B.240 | T/N.1;T.1;B.1;B.2;B.3;B.5;B.6;B.10;B.11;B.44;B.45;B.240 | 5 | 5 |
| 19T0241 | 19T0241 | | 2019 | FLI | | Rems-Murr-Kreis | Lepus europaeus | B.6 | B.45 | B.240 | T/N.1;T.1;B.1;B.2;B.3;B.5;B.6;B.10;B.11;B.44;B.45;B.240 | 5 | 5 |
| 19T0279 | 19T0279 | | 2019 | FLI | Ludwigsburg | Ludwigsburg | Lepus europaeus | B.6 | B.63 | B.63 | T/N.1;T.1;B.1;B.2;B.3;B.5;B.6;B.10;B.11;B.44;B.228;B.46;B.47;B.63 | 6 | 6 |
| 19T0366 | 19T0366 | | 2019 | FLI | | Rems-Murr-Kreis | Lepus europaeus | B.6 | B.61 | B.284 | T/N.1;T.1;B.1;B.2;B.3;B.5;B.6;B.10;B.11;B.44;B.45;B.50;B.61;B.284 | 8 | 2 |
| 19T0443 | 19T0443 | | 2019 | FLI | Boeblingen | Boeblingen | Lepus europaeus | B.6 | B.61 | B.283 | T/N.1;T.1;B.1;B.2;B.3;B.5;B.6;B.10;B.11;B.44;B.45;B.50;B.61;B.283 | | 7 |
| 19T0444 | 19T0444 | | 2019 | FLI | Schwaebisch Hall | Schwaebisch Hall | Lepus europaeus | B.6 | B.45 | B.240 | T/N.1;T.1;B.1;B.2;B.3;B.5;B.6;B.10;B.11;B.44;B.45;B.240 | | |
| 19T0454 | 19T0454 | | 2019 | FLI | Boeblingen | Boeblingen | Lepus europaeus | B.6 | B.63 | B.63 | T/N.1;T.1;B.1;B.2;B.3;B.5;B.6;B.10;B.11;B.44;B.228;B.46;B.47;B.63 | 6 | 6 |
| 20T0086 | 20T0086 | | 2020 | FLI | Alfdorf | Rems-Murr-Kreis | Lepus europaeus | B.6 | B.61 | B.283 | T/N.1;T.1;B.1;B.2;B.3;B.5;B.6;B.10;B.11;B.44;B.45;B.50;B.61;B.283 | | 7 |
| 20T0088 | 20T0088 | | 2020 | FLI | Schorndorf-Weiler | Rems-Murr-Kreis | Lepus europaeus | B.6 | B.61 | B.283 | T/N.1;T.1;B.1;B.2;B.3;B.5;B.6;B.10;B.11;B.44;B.45;B.50;B.61;B.283 | | 7 |
| 20T0142 | 20T0142 | | 2020 | FLI | Schrozberg | Schwaebisch Hall | Lepus europaeus | B.6 | B.49 | B.266 | T/N.1;T.1;B.1;B.2;B.3;B.5;B.6;B.10;B.11;B.44;B.45;B.49;B.266 | 15 | 1 |
| 20T0145 | 20T0145 | | 2020 | FLI | Illingen | Ludwigsburg | Lepus europaeus | B.6 | B.61 | B.281 | T/N.1;T.1;B.1;B.2;B.3;B.5;B.6;B.10;B.11;B.44;B.45;B.50;B.61;B.281 | | 2 |
| 20T0179 | 20T0179 | | 2020 | FLI | Winterbach | Rems-Murr-Kreis | Lepus europaeus | B.6 | B.61 | B.284 | T/N.1;T.1;B.1;B.2;B.3;B.5;B.6;B.10;B.11;B.44;B.45;B.50;B.61;B.284 | 8 | 2 |
| 20T0202 | 20T0202 | | 2020 | FLI | Kressberg-Bergbronn | Schwaebisch Hall | Lepus europaeus | B.12 | B.33 | B.106 | T/N.1;T.1;B.1;B.2;B.3;B.5;B.12;B.72;B.13;B.26;B.42;B.168;B.66;B.33;B.103;B.106 | | |
| 20T0233 | 20T0233 | | 2020 | FLI | Winterbach | Rems-Murr-Kreis | Lepus europaeus | B.6 | B.61 | B.284 | T/N.1;T.1;B.1;B.2;B.3;B.5;B.6;B.10;B.11;B.44;B.45;B.50;B.61;B.284 | 8 | 2 |

| ID | Strain Name | Pubmed. BioProject. Accession | Year | Institute* | City | County (district) | Host | Major Clade | SubCan Snper1 | SubCan Snper2 | SNP_Path | Max SNPs_1 | Max SNPs_5 |
|---|---|---|---|---|---|---|---|---|---|---|---|---|---|
| 20T0239 | 20T0239 | | 2020 | FLI | Schrozberg | Schwaebisch Hall | Lepus europaeus | B.6 | B.45 | B.248 | T/N.1;T.1;B.1;B.2;B.3;B.5;B.6;B.10;B.11;B.44;B.45;B.247;B.248 | 17 | 15 |
| 20T0242 | 20T0242 | | 2020 | FLI | | Main-Tauber | Lepus europaeus | B.6 | B.46 | B.46 | T/N.1;T.1;B.1;B.2;B.3;B.5;B.6;B.10;B.11;B.44;B.228;B.46 | | |
| 20T0245 | 20T0245 | | 2020 | FLI | Buehlerzell | Schwaebisch Hall | Lepus europaeus | B.6 | B.49 | B.49 | T/N.1;T.1;B.1;B.2;B.3;B.5;B.6;B.10;B.11;B.44;B.45;B.49 | | 1 |
| 20T0246 | 20T0246 | | 2020 | FLI | | Schwaebisch Hall | Lepus europaeus | B.6 | B.62 | B.110 | T/N.1;T.1;B.1;B.2;B.3;B.5;B.6;B.10;B.11;B.44;B.45;B.51;B.62;B.110 | | 13 |
| 20T0249 | 20T0249 | | 2020 | | | Calw | Lepus europaeus | B.6 | B.61 | B.288 | T/N.1;T.1;B.1;B.2;B.3;B.5;B.6;B.10;B.11;B.44;B.45;B.50;B.61;B.285;B.287;B.288 | | |
| 21T0014 | 21T0014 | | 2021 | FLI | Konstanz | | Lepus europaeus | B.6 | B.59 | B.59 | T/N.1;T.1;B.1;B.2;B.3;B.5;B.6;B.10;B.11;B.44;B.45;B.50;B.59 | | 9 |
| 21T0015 | 21T0015 | | 2021 | FLI | | | Cyanistes caeruleus | B.6 | B.49 | B.49 | T/N.1;T.1;B.1;B.2;B.3;B.5;B.6;B.10;B.11;B.44;B.45;B.49 | | |
| 21T0101 | 21T0101 | | 2021 | FLI | Backnang | Rems-Murr-Kreis | Lepus europaeus | B.6 | B.63 | B.63 | T/N.1;T.1;B.1;B.2;B.3;B.5;B.6;B.10;B.11;B.44;B.228;B.46;B.47;B.63 | 18 | 8 |
| 22T0158 | 22T0158 | | 2022 | FLI | Heilbronn | Heilbronn | Lepus europaeus | B.6 | B.45 | B.87 | T/N.1;T.1;B.1;B.2;B.3;B.5;B.6;B.10;B.11;B.44;B.45;B.87 | | 3 |
| 22T0160 | 22T0160 | | 2022 | FLI | | Schwaebisch Hall | Lepus europaeus | B.6 | B.61 | B.279 | T/N.1;T.1;B.1;B.2;B.3;B.5;B.6;B.10;B.11;B.44;B.45;B.50;B.61;B.279 | | |
| A1475 | A1475 | | 2019 | RKI** | | Ravensburg | Homo sapiens | B.6 | B.45 | B.91 | T/N.1;T.1;B.1;B.2;B.3;B.5;B.6;B.10;B.11;B.44;B.45;B.91 | | |
| A1480 | A1480 | | 2019 | RKI** | | Emmendingen | Homo sapiens | B.6 | B.61 | B.279 | T/N.1;T.1;B.1;B.2;B.3;B.5;B.6;B.10;B.11;B.44;B.45;B.50;B.61;B.279 | | |
| A1487 | A1487 | | 2019 | RKI** | | Breisgau-Hoch-schwarzwald | Homo sapiens | B.6 | B.49 | B.49 | T/N.1;T.1;B.1;B.2;B.3;B.5;B.6;B.10;B.11;B.44;B.45;B.49 | | |
| A1489 | A1489 | | 2019 | RKI** | | Esslingen | Homo sapiens | B.6 | B.61 | B.281 | T/N.1;T.1;B.1;B.2;B.3;B.5;B.6;B.10;B.11;B.44;B.45;B.50;B.61;B.281 | | |
| A1574 | A1574 | | 2019 | RKI** | | Alb-Donau-Kreis | Homo sapiens | B.6 | B.45 | B.45 | T/N.1;T.1;B.1;B.2;B.3;B.5;B.6;B.10;B.11;B.44;B.45 | | |
| A1586 | A1586 | | 2019 | RKI** | | Breisgau-Hochschwarzwald | Homo sapiens | B.6 | B.49 | B.49 | T/N.1;T.1;B.1;B.2;B.3;B.5;B.6;B.10;B.11;B.44;B.45;B.49 | | 10 |
| A1666 | A1666 | | 2020 | RKI** | | Main-Tauber-Kreis | Homo sapiens | B.6 | B.45 | B.248 | T/N.1;T.1;B.1;B.2;B.3;B.5;B.6;B.10;B.11;B.44;B.45;B.247;B.248 | | |
| A1667 | A1667 | | 2020 | RKI** | | Heilbronn | Homo sapiens | B.6 | B.45 | B.87 | T/N.1;T.1;B.1;B.2;B.3;B.5;B.6;B.10;B.11;B.44;B.45;B.87 | 13 | 3 |
| A1691 | A1691 | | 2020 | RKI** | | Breisgau-Hoch-schwarzwald | Homo sapiens | B.6 | B.45 | B.87 | T/N.1;T.1;B.1;B.2;B.3;B.5;B.6;B.10;B.11;B.44;B.45;B.87 | | 1 |
| A1761 | A1761 | | 2021 | RKI** | | Ravensburg | Homo sapiens | B.12 | B.33 | B.103 | T/N.1;T.1;B.1;B.2;B.3;B.5;B.12;B.72;B.13;B.26;B.42;B.168;B.66;B.33;B.103 | | |
| A1772 | A1772 | | 2021 | RKI** | | Ortenaukreis | Homo sapiens | B.6 | B.45 | B.305 | T/N.1;T.1;B.1;B.2;B.3;B.5;B.6;B.10;B.11;B.44;B.45;B.89;B.305 | | |
| A1817 | A1817 | | 2021 | RKI** | | Rhein-Neckar-Kreis | Homo sapiens | B.6 | B.45 | B.45 | T/N.1;T.1;B.1;B.2;B.3;B.5;B.6;B.10;B.11;B.44;B.45 | 7 | 1 |
| A1871 | A1871 | | 2021 | RKI** | | Alb-Donau-Kreis | Homo sapiens | B.6 | B.63 | B.63 | T/N.1;T.1;B.1;B.2;B.3;B.5;B.6;B.10;B.11;B.44;B.228;B.46;B.47;B.63 | | 8 |

| ID | Strain Name | Pubmed. BioProject. Accession | Year | Institute* | City | County (district) | Host | Major Clade | SubCan Snper1 | SubCan Snper2 | SNP_Path | Max SNPs_ 1 | Max SNPs_ 5 |
|---|---|---|---|---|---|---|---|---|---|---|---|---|---|
| A1893 | A1893 | | 2021 | RKI** | | Ravensburg | Homo sapiens | B.6 | B.63 | B.63 | T/N.1;T.1;B.1;B.2;B.3;B.5;B.6;B.10;B.11;B.44;B.228;B.46;B.47;B .63 | 18 | 8 |
| A1964 | A1964 | | 2022 | RKI** | | Ravensburg | Homo sapiens | B.6 | B.45 | B.91 | T/N.1;T.1;B.1;B.2;B.3;B.5;B.6;B.10;B.11;B.44;B.45;B.91 | | 1 |
| A1985 | A1985 | | 2022 | RKI** | | Ostalbkreis | Homo sapiens | B.12 | B.33 | B.182 | T/N.1;T.1;B.1;B.2;B.3;B.5;B.12;B.72;B.13;B.26;B.42;B.168;B.66; B.33;B.103;B.182 | | |
| ERR3555 285 | A936 | PRJEB33006 | 2017 | RKI | | Ortenaukreis | Homo sapiens | B.6 | B.49 | B.49 | T/N.1;T.1;B.1;B.2;B.3;B.5;B.6;B.10;B.11;B.44;B.45;B.49 | 3 | 1 |
| ERR3555 286 | A981 | PRJEB33006 | 2017 | RKI | | Tuebingen | Homo sapiens | B.6 | B.49 | B.49 | T/N.1;T.1;B.1;B.2;B.3;B.5;B.6;B.10;B.11;B.44;B.45;B.49 | 3 | 1 |
| ERR3555 295 | A1158 | PRJEB33006 | 2018 | RKI | | Ostalbkreis | Homo sapiens | B.6 | B.61 | B.287 | T/N.1;T.1;B.1;B.2;B.3;B.5;B.6;B.10;B.11;B.44;B.45;B.50;B.61;B. 285;B.287 | | |
| ERR3555 297 | A1174 | PRJEB33006 | 2018 | RKI | Karlsruhe | Karlsruhe | Homo sapiens | B.6 | B.45 | B.45 | T/N.1;T.1;B.1;B.2;B.3;B.5;B.6;B.10;B.11;B.44;B.45 | 3 | 1 |
| ERR3555 301 | A9222 | PRJEB33006 | 2017 | RKI | | Alb-Donau-Kreis | Homo sapiens | B.6 | B.45 | B.259 | T/N.1;T.1;B.1;B.2;B.3;B.5;B.6;B.10;B.11;B.44;B.45;B.259 | | |
| ERR3555 305 | A660 | PRJEB33006 | 2015 | RKI | | | Homo sapiens | B.6 | B.45 | B.45 | T/N.1;T.1;B.1;B.2;B.3;B.5;B.6;B.10;B.11;B.44;B.45 | | |
| ERR3555 308 | A797 | PRJEB33006 | 2016 | RKI | | Ortenaukreis | Homo sapiens | B.12 | B.34 | B.34 | T/N.1;T.1;B.1;B.2;B.3;B.5;B.12;B.72;B.13;B.26;B.42;B.168;B.66; B.33;B.34 | | |

*FLI: Friedrich-Loeffler-Institute, RKI: Robert-Koch-Institute
**unpublished

**Appendix Table 2.** Quality measures of raw sequencing data and assemblies using in this study

| ID | Common name | perc_Q30 | Reference genome size | Total reads | Theoretical coverage | 1st Match Genus | % 1st Match Genus | 1st Match Species | Amplicon size RD-1 | %1st Match Species | # contigs | Assembly Size bp | N50 | GC (%) |
|---|---|---|---|---|---|---|---|---|---|---|---|---|---|---|
| 08T0014 | 08T0014 | 0,8329 | 1919424 | 21519832 | 1401 | Francisella | 99,74 | F. tularensis | 924 | 99,28 | 99 | 1783422 | 26890 | 32,25 |
| 08T0015 | 08T0015 | 0,845068 | 1919424 | 1430820 | 94 | Francisella | 97,74 | F. tularensis | 924 | 90,58 | 48 | 1876439 | 100618 | 32,45 |
| 09T0109 | 09T0109 | 0,86591 | 1919424 | 4212020 | 247 | Francisella | 96,95 | F. tularensis | 924 | 89,51 | 208 | 1771092 | 13573 | 32,38 |
| 09T0146 | 09T0146 | 0,854123 | 1919424 | 4944206 | 301 | Francisella | 97,87 | F. tularensis | 924 | 91,87 | 168 | 1772532 | 19291 | 32,3 |
| 10T0131 | 10T0131 | 0,908184 | 1919424 | 495492 | 60 | Francisella | 99,54 | F. tularensis | 924 | 98,1 | 97 | 1787965 | 27360 | 32,17 |
| 10T0134 | 10T0134 | 0,924141 | 1919424 | 979584 | 116 | Francisella | 99,19 | F. tularensis | 924 | 97,39 | 101 | 1789952 | 26622 | 32,17 |
| 10T0145 | 10T0145 | 0,851997 | 1919424 | 1098642 | 125 | Francisella | 99,15 | F. tularensis | 924 | 97,16 | 99 | 1787375 | 26987 | 32,17 |
| 10T0162 | 10T0162 | 0,867908 | 1919424 | 1712182 | 213 | Francisella | 99,4 | F. tularensis | 924 | 98,25 | 98 | 1787398 | 26986 | 32,17 |
| 10T0165 | 10T0165 | 0,867859 | 1919424 | 1209382 | 155 | Francisella | 99,56 | F. tularensis | 924 | 98,57 | 99 | 1787973 | 26986 | 32,17 |
| 10T0167 | 10T0167 | 0,882922 | 1919424 | 3835500 | 249 | Francisella | 99,72 | F. tularensis | 924 | 99,28 | 97 | 1783308 | 27534 | 32,25 |
| 10T0168 | 10T0168 | 0,872834 | 1919424 | 375196 | 52 | Francisella | 99,7 | F. tularensis | 924 | 98,75 | 122 | 1784280 | 22826 | 32,2 |
| 10T0188 | 10T0188 | 0,882563 | 1919424 | 3835500 | 249 | Francisella | 99,75 | F. tularensis | 924 | 99,09 | 99 | 1783495 | 26890 | 32,25 |
| 10T0189 | 10T0189 | 0,908807 | 1919424 | 1052868 | 135 | Francisella | 99,71 | F. tularensis | 924 | 98,71 | 99 | 1787592 | 26986 | 32,17 |
| 10T0195 | 10T0195 | 0,985098 | 1919424 | 450120 | 47 | Francisella | 99,56 | F. tularensis | 924 | 97,84 | 101 | 1788251 | 26986 | 32,18 |
| 11T0041 | 11T0041 | 0,907534 | 1919424 | 464562 | 54 | Francisella | 99,65 | F. tularensis | 924 | 98,22 | 100 | 1787589 | 26622 | 32,17 |
| 12T0023 | 12T0023 | 0,847103 | 1919424 | 892296 | 102 | Francisella | 99,41 | F. tularensis | 924 | 97,94 | 98 | 1787870 | 27087 | 32,17 |
| 12T0048 | 12T0048 | 0,923754 | 1919424 | 1318512 | 153 | Francisella | 99,58 | F. tularensis | 924 | 98,14 | 100 | 1787218 | 26622 | 32,17 |
| 13T0036 | 13T0036 | 0,929701 | 1919424 | 1049012 | 107 | Francisella | 99,48 | F. tularensis | 924 | 97,47 | 99 | 1787515 | 26986 | 32,17 |
| 13T0063 | 13T0063 | 0,903909 | 1919424 | 458154 | 49 | Francisella | 99,13 | F. tularensis | 924 | 97,08 | 99 | 1787376 | 26987 | 32,17 |
| 13T0064 | 13T0064 | 0,909839 | 1919424 | 473088 | 57 | Francisella | 99,66 | F. tularensis | 924 | 98,5 | 100 | 1787512 | 26622 | 32,17 |
| 13T0110 | 13T0110 | 0,916953 | 1919424 | 714542 | 80 | Francisella | 99,44 | F. tularensis | 924 | 97,77 | 101 | 1788356 | 26986 | 32,17 |
| 14T0026 | 14T0026 | 0,915765 | 1919424 | 515508 | 60 | Francisella | 99,72 | F. tularensis | 924 | 98,56 | 103 | 1787562 | 25319 | 32,17 |

| ID | Common name | perc_Q30 | Reference genome size | Total reads | Theoretical coverage | 1st Match Genus | % 1st Match Genus | 1st Match Species | Amplicon size RD-1 | %1st Match Species | # contigs | Assembly Size bp | N50 | GC (%) |
|---|---|---|---|---|---|---|---|---|---|---|---|---|---|---|
| 14T0098 | 14T0098 | 0,913986 | 1919424 | 624540 | 72 | Francisella | 99,49 | F. tularensis | 924 | 98,03 | 99 | 1787354 | 26986 | 32,17 |
| 15T0224 | 15T0224 | 0,930571 | 1919424 | 3835500 | 251 | Francisella | 99,91 | F. tularensis | 924 | 99,41 | 94 | 1778156 | 29741 | 32,21 |
| 15T0767 | 15T0767 | 0,85033 | 1919424 | 537438 | 49 | Francisella | 98,65 | F. tularensis | 924 | 95,32 | 121 | 1782446 | 22825 | 32,2 |
| 15T0808 | 15T0808 | 0,917611 | 1919424 | 3835500 | 251 | Francisella | 99,06 | F. tularensis | 924 | 98,56 | 98 | 1777634 | 26890 | 32,21 |
| 16T0007 | 16T0007 | 0,88646 | 1919424 | 1516218 | 172 | Francisella | 99,62 | F. tularensis | 924 | 98,3 | 103 | 1787398 | 25319 | 32,17 |
| 16T0022 | 16T0022 | 0,894467 | 1919424 | 10751036 | 845 | Francisella | 99,6 | F. tularensis | 924 | 99,21 | 97 | 1785036 | 27598 | 32,19 |
| 16T0051 | 16T0051 | 0,912257 | 1919424 | 17114668 | 1346 | Francisella | 99,87 | F. tularensis | 924 | 99,46 | 98 | 1783817 | 26954 | 32,18 |
| 16T0052 | 16T0052 | 0,887631 | 1919424 | 13623366 | 1071 | Francisella | 99,84 | F. tularensis | 924 | 99,42 | 98 | 1784306 | 26954 | 32,18 |
| 16T0053 | 16T0053 | 0,907799 | 1919424 | 18131184 | 1426 | Francisella | 99,83 | F. tularensis | 924 | 99,39 | 98 | 1783823 | 26954 | 32,18 |
| 16T0054 | 16T0054 | 0,910388 | 1919424 | 15281316 | 1202 | Francisella | 99,85 | F. tularensis | 924 | 99,41 | 100 | 1785367 | 26955 | 32,19 |
| 16T0621 | 16T0621 | 0,901251 | 1919424 | 16002990 | 1258 | Francisella | 99,76 | F. tularensis | 924 | 99,35 | 97 | 1785224 | 27598 | 32,19 |
| 16T0622 | 16T0622 | 0,891143 | 1919424 | 10534434 | 828 | Francisella | 98,92 | F. tularensis | 924 | 98,56 | 103 | 1791701 | 27598 | 32,24 |
| 16T0623 | 16T0623 | 0,905518 | 1919424 | 17764264 | 1397 | Francisella | 99,82 | F. tularensis | 924 | 99,4 | 97 | 1784081 | 27329 | 32,18 |
| 16T1168 | 16T1168 | 0,910365 | 1919424 | 16784238 | 1320 | Francisella | 99,87 | F. tularensis | 924 | 99,47 | 98 | 1785689 | 26955 | 32,18 |
| 16T1169 | 16T1169 | 0,878727 | 1919424 | 12564418 | 988 | Francisella | 99,77 | F. tularensis | 924 | 99,38 | 98 | 1786059 | 26955 | 32,18 |
| 16T1176 | 16T1176 | 0,910066 | 1919424 | 17358442 | 1365 | Francisella | 99,78 | F. tularensis | 924 | 99,38 | 99 | 1787169 | 27594 | 32,19 |
| 17T1202 | 17T1202 | 0,914813 | 1919424 | 229874 | 30 | Francisella | 99,56 | F. tularensis | 924 | 98,42 | 109 | 1785548 | 23699 | 32,18 |
| 17T1203 | 17T1203 | 0,919931 | 1919424 | 771518 | 76 | Francisella | 98,82 | F. tularensis | 924 | 95,91 | 101 | 1787561 | 26617 | 32,17 |
| 17T1415 | 17T1415 | 0,846382 | 1919424 | 1118628 | 146 | Francisella | 99,59 | F. tularensis | 924 | 98,46 | 101 | 1787534 | 26622 | 32,17 |
| 17T1542 | 17T1542 | 0,908628 | 1919424 | 893928 | 115 | Francisella | 99,55 | F. tularensis | 924 | 98,47 | 97 | 1787881 | 27630 | 32,17 |
| 18T0029 | 18T0029 | 0,847484 | 1919424 | 1510702 | 194 | Francisella | 99,59 | F. tularensis | 924 | 98,49 | 98 | 1787413 | 26986 | 32,17 |
| 18T0158 | 18T0158 | 0,848574 | 1919424 | 896806 | 101 | Francisella | 99,2 | F. tularensis | 924 | 97,15 | 99 | 1789140 | 26987 | 32,17 |
| 18T0161 | 18T0161 | 0,850026 | 1919424 | 973790 | 123 | Francisella | 99,52 | F. tularensis | 924 | 98,33 | 99 | 1787459 | 26986 | 32,17 |
| 18T0163 | 18T0163 | 0,907138 | 1919424 | 3411906 | 255 | Francisella | 98,62 | F. tularensis | 924 | 94,52 | 99 | 1780489 | 26914 | 32,2 |
| 18T0164 | 18T0164 | 0,859399 | 1919424 | 1579162 | 203 | Francisella | 99,59 | F. tularensis | 924 | 98,52 | 99 | 1787755 | 26986 | 32,17 |
| 18T0195 | 18T0195 | 0,818289 | 1919424 | 709704 | 85 | Francisella | 99,45 | F. tularensis | 924 | 98,02 | 111 | 1787487 | 25319 | 32,17 |
| 18T0198 | 18T0198 | 0,865874 | 1919424 | 847686 | 95 | Francisella | 99,2 | F. tularensis | 924 | 97,3 | 100 | 1787484 | 26622 | 32,17 |
| 18T2158 | 18T2158 | 0,880439 | 1919424 | 968274 | 107 | Francisella | 99,25 | F. tularensis | 924 | 97,37 | 101 | 1786368 | 26987 | 32,18 |
| 18T2163 | 18T2163 | 0,859235 | 1919424 | 505346 | 58 | Francisella | 99,23 | F. tularensis | 924 | 97,59 | 121 | 1785256 | 23379 | 32,19 |
| 18T2170 | 18T2170 | 0,867952 | 1919424 | 835832 | 96 | Francisella | 99,18 | F. tularensis | 924 | 97,34 | 108 | 1786807 | 24075 | 32,17 |
| 19T0002 | 19T0002 | 0,88525 | 1919424 | 1332472 | 148 | Francisella | 98,88 | F. tularensis | 924 | 96,71 | 101 | 1787520 | 26622 | 32,17 |
| 19T0003 | 19T0003 | 0,871297 | 1919424 | 840420 | 105 | Francisella | 99,19 | F. tularensis | 924 | 97,68 | 97 | 1787881 | 27630 | 32,17 |
| 19T0037 | 19T0037 | 0,859935 | 1919424 | 649248 | 82 | Francisella | 99,3 | F. tularensis | 924 | 97,85 | 99 | 1787711 | 27087 | 32,17 |
| 19T0045 | 19T0045 | 0,840195 | 1919424 | 454826 | 60 | Francisella | 99,47 | F. tularensis | 924 | 98,45 | 99 | 1787771 | 26986 | 32,17 |
| 19T0046 | 19T0046 | 0,878162 | 1919424 | 696828 | 88 | Francisella | 99,18 | F. tularensis | 924 | 97,85 | 100 | 1787843 | 26986 | 32,17 |
| 19T0051 | 19T0051 | 0,919123 | 1919424 | 558894 | 57 | Francisella | 98,51 | F. tularensis | 924 | 95,56 | 102 | 1787166 | 26622 | 32,17 |
| 19T0062 | 19T0062 | 0,931185 | 1919424 | 351550 | 41 | Francisella | 99,33 | F. tularensis | 924 | 97,73 | 99 | 1787768 | 26986 | 32,17 |
| 19T0063 | 19T0063 | 0,929632 | 1919424 | 593624 | 66 | Francisella | 99,27 | F. tularensis | 924 | 97,58 | 99 | 1787513 | 26986 | 32,17 |
| 19T0098 | 19T0098 | 0,932753 | 1919424 | 508430 | 60 | Francisella | 99,49 | F. tularensis | 924 | 98,01 | 99 | 1787491 | 26986 | 32,17 |
| 19T0118 | 19T0118 | 0,929995 | 1919424 | 1022282 | 119 | Francisella | 99,25 | F. tularensis | 924 | 97,45 | 99 | 1788688 | 26987 | 32,17 |
| 19T0120 | 19T0120 | 0,939341 | 1919424 | 1365114 | 154 | Francisella | 99,45 | F. tularensis | 924 | 97,83 | 100 | 1788761 | 26622 | 32,17 |
| 19T0160 | 19T0160 | 0,933845 | 1919424 | 464870 | 44 | Francisella | 98,87 | F. tularensis | 924 | 95,94 | 107 | 1785050 | 25319 | 32,18 |
| 19T0161 | 19T0161 | 0,914462 | 1919424 | 489200 | 56 | Francisella | 99,27 | F. tularensis | 924 | 97,37 | 113 | 1787952 | 23378 | 32,17 |
| 19T0165 | 19T0165 | 0,948129 | 1919424 | 666492 | 77 | Francisella | 99,46 | F. tularensis | 924 | 97,93 | 99 | 1787083 | 26622 | 32,17 |
| 19T0191 | 19T0191 | 0,948198 | 1919424 | 495460 | 57 | Francisella | 99,39 | F. tularensis | 924 | 97,95 | 100 | 1787516 | 26986 | 32,17 |
| 19T0194 | 19T0194 | 0,944245 | 1919424 | 403108 | 47 | Francisella | 99,4 | F. tularensis | 924 | 98,01 | 103 | 1787536 | 26986 | 32,17 |
| 19T0240 | 19T0240 | 0,937387 | 1919424 | 849752 | 99 | Francisella | 99,44 | F. tularensis | 924 | 98,04 | 99 | 1787455 | 26986 | 32,17 |
| 19T0241 | 19T0241 | 0,94434 | 1919424 | 990612 | 117 | Francisella | 99,39 | F. tularensis | 924 | 98 | 99 | 1787629 | 26986 | 32,17 |

| ID | Common name | perc_Q30 | Reference genome size | Total reads | Theoretical coverage | 1st Match Genus | % 1st Match Genus | 1st Match Species | Amplicon size RD-1 | %1st Match Species | # contigs | Assembly Size bp | N50 | GC (%) |
|---|---|---|---|---|---|---|---|---|---|---|---|---|---|---|
| 19T0279 | 19T0279 | 0,941236 | 1919424 | 289402 | 35 | Francisella | 99,26 | F. tularensis | 924 | 97,67 | 100 | 1788414 | 26986 | 32,17 |
| 19T0366 | 19T0366 | 0,952727 | 1919424 | 1006390 | 107 | Francisella | 98,96 | F. tularensis | 924 | 96,83 | 99 | 1787653 | 26986 | 32,17 |
| 19T0443 | 19T0443 | 0,94469 | 1919424 | 795124 | 95 | Francisella | 99,24 | F. tularensis | 924 | 97,53 | 100 | 1788314 | 26986 | 32,17 |
| 19T0444 | 19T0444 | 0,946427 | 1919424 | 840572 | 97 | Francisella | 99,08 | F. tularensis | 924 | 97,17 | 99 | 1787539 | 26986 | 32,17 |
| 19T0454 | 19T0454 | 0,94931 | 1919424 | 745994 | 85 | Francisella | 99,23 | F. tularensis | 924 | 97,29 | 99 | 1787461 | 26986 | 32,17 |
| 20T0086 | 20T0086 | 0,884593 | 1919424 | 1355142 | 163 | Francisella | 99,58 | F. tularensis | 924 | 98,06 | 101 | 1787668 | 26622 | 32,17 |
| 20T0088 | 20T0088 | 0,882977 | 1919424 | 1210092 | 151 | Francisella | 99,63 | F. tularensis | 924 | 98,28 | 105 | 1787434 | 26622 | 32,17 |
| 20T0142 | 20T0142 | 0,904315 | 1919424 | 1413610 | 151 | Francisella | 99,47 | F. tularensis | 924 | 97,43 | 100 | 1787547 | 26986 | 32,17 |
| 20T0145 | 20T0145 | 0,927147 | 1919424 | 1116200 | 113 | Francisella | 99 | F. tularensis | 924 | 96,14 | 100 | 1787501 | 26986 | 32,17 |
| 20T0179 | 20T0179 | 0,939465 | 1919424 | 1510160 | 162 | Francisella | 99,42 | F. tularensis | 924 | 97,44 | 102 | 1788248 | 26622 | 32,17 |
| 20T0202 | 20T0202 | 0,933466 | 1919424 | 1383614 | 154 | Francisella | 99,51 | F. tularensis | 924 | 97,79 | 100 | 1789458 | 26987 | 32,17 |
| 20T0233 | 20T0233 | 0,936012 | 1919424 | 1159010 | 125 | Francisella | 99,46 | F. tularensis | 924 | 97,47 | 99 | 1787476 | 26986 | 32,17 |
| 20T0239 | 20T0239 | 0,927745 | 1919424 | 1408126 | 141 | Francisella | 98,97 | F. tularensis | 924 | 96,12 | 102 | 1787425 | 27087 | 32,17 |
| 20T0242 | 20T0242 | 0,936004 | 1919424 | 1444474 | 158 | Francisella | 99,45 | F. tularensis | 924 | 97,53 | 100 | 1787700 | 26986 | 32,17 |
| 20T0245 | 20T0245 | 0,931795 | 1919424 | 1199176 | 137 | Francisella | 99,57 | F. tularensis | 924 | 97,98 | 99 | 1787457 | 26986 | 32,17 |
| 20T0246 | 20T0246 | 0,909998 | 1919424 | 994354 | 110 | Francisella | 99,29 | F. tularensis | 924 | 97,05 | 101 | 1787519 | 25319 | 32,17 |
| 20T0249 | 20T0249 | 0,929394 | 1919424 | 1526202 | 163 | Francisella | 99,39 | F. tularensis | 924 | 97,36 | 99 | 1787501 | 26986 | 32,17 |
| 21T0014 | 21T0014 | 0,910039 | 1919424 | 947358 | 104 | Francisella | 99,32 | F. tularensis | 924 | 97,35 | 100 | 1787792 | 26986 | 32,17 |
| 21T0015 | 21T0015 | 0,924485 | 1919424 | 722718 | 82 | Francisella | 99,29 | F. tularensis | 924 | 97,44 | 99 | 1788326 | 26986 | 32,17 |
| 21T0101 | 21T0101 | 0,909569 | 1919424 | 1086854 | 122 | Francisella | 99,28 | F. tularensis | 924 | 97,67 | 99 | 1787455 | 26986 | 32,17 |
| 22T0158 | 22T0158 | 0,898056 | 1919424 | 904542 | 103 | Francisella | 99,13 | F. tularensis | 924 | 97,06 | 104 | 1788264 | 26622 | 32,17 |
| 22T0160 | 22T0160 | 0,877948 | 1919424 | 1155732 | 125 | Francisella | 99,06 | F. tularensis | 924 | 96,99 | 101 | 1787547 | 25319 | 32,17 |
| A1475 | A1475 | 0,908671 | 1919424 | 2296304 | 282 | Francisella | 99,77 | F. tularensis | 924 | 97,18 | 100 | 1787473 | 26622 | 32,17 |
| A1480 | A1480 | 0,903298 | 1919424 | 2068024 | 279 | Francisella | 99,87 | F. tularensis | 924 | 97,89 | 101 | 1787366 | 25319 | 32,17 |
| A1487 | A1487 | 0,905523 | 1919424 | 1999958 | 205 | Francisella | 99,52 | F. tularensis | 924 | 97,22 | 104 | 1787520 | 26622 | 32,17 |
| A1489 | A1489 | 0,903301 | 1919424 | 2259708 | 276 | Francisella | 99,78 | F. tularensis | 924 | 97,26 | 102 | 1787251 | 26986 | 32,17 |
| A1574 | A1574 | 0,919836 | 1919424 | 2215730 | 247 | Francisella | 99,74 | F. tularensis | 924 | 97,28 | 101 | 1787358 | 26622 | 32,17 |
| A1586 | A1586 | 0,906455 | 1919424 | 1983348 | 190 | Francisella | 99,41 | F. tularensis | 924 | 96,79 | 100 | 1787496 | 26622 | 32,17 |
| A1666 | A1666 | 0,911837 | 1919424 | 2070744 | 185 | Francisella | 99,32 | F. tularensis | 924 | 96,28 | 110 | 1787735 | 25627 | 32,17 |
| A1667 | A1667 | 0,956283 | 1919424 | 3252902 | 330 | Francisella | 99,67 | F. tularensis | 924 | 97,67 | 110 | 1785716 | 24037 | 32,18 |
| A1691 | A1691 | 0,914437 | 1919424 | 2354084 | 201 | Francisella | 99,33 | F. tularensis | 924 | 96,21 | 101 | 1782938 | 26938 | 32,19 |
| A1761 | A1761 | 0,869957 | 1919424 | 2572432 | 341 | Francisella | 99,87 | F. tularensis | 924 | 98,44 | 101 | 1788955 | 25532 | 32,17 |
| A1772 | A1772 | 0,953461 | 1919424 | 3503162 | 297 | Francisella | 99,31 | F. tularensis | 924 | 95,67 | 99 | 1783155 | 26574 | 32,19 |
| A1817 | A1817 | 0,910867 | 1919424 | 2743958 | 337 | Francisella | 99,83 | F. tularensis | 924 | 97,95 | 99 | 1787597 | 26986 | 32,17 |
| A1871 | A1871 | 0,848014 | 1919424 | 3002474 | 361 | Francisella | 99,76 | F. tularensis | 924 | 97,39 | 100 | 1788109 | 26986 | 32,18 |
| A1893 | A1893 | 0,825448 | 1919424 | 2436686 | 324 | Francisella | 99,88 | F. tularensis | 924 | 98,16 | 102 | 1787443 | 26622 | 32,17 |
| A1964 | A1964 | 0,838963 | 1919424 | 2988750 | 398 | Francisella | 99,83 | F. tularensis | 924 | 98,05 | 174 | 1767999 | 16292 | 32,28 |
| A1985 | A1985 | 0,787212 | 1919424 | 1252624 | 186 | Francisella | 99,9 | F. tularensis | 924 | 99,25 | 101 | 1787238 | 25293 | 32,18 |
| ERR3555285 | A936 | 0,808932 | 1919424 | 1264112 | 198 | Francisella | 99,49 | F. tularensis | 924 | 98,16 | 106 | 1787944 | 23411 | 32,19 |
| ERR3555286 | A981 | 0,821471 | 1919424 | 848396 | 133 | Francisella | 99,45 | F. tularensis | 924 | 98,18 | 119 | 1795371 | 23411 | 32,2 |
| ERR3555295 | A1158 | 0,953942 | 1919424 | 2111968 | 238 | Francisella | 99,58 | F. tularensis | 924 | 98,2 | 114 | 1784242 | 24075 | 32,18 |
| ERR3555297 | A1174 | 0,950619 | 1919424 | 2296300 | 247 | Francisella | 99,66 | F. tularensis | 924 | 98,06 | 114 | 1784623 | 23379 | 32,19 |
| ERR3555301 | A9222 | 0,945972 | 1919424 | 2457960 | 288 | Francisella | 99,73 | F. tularensis | 924 | 98,48 | 105 | 1789309 | 26622 | 32,19 |
| ERR3555305 | A660 | 0,894455 | 1919424 | 1872754 | 239 | Francisella | 99,64 | F. tularensis | 924 | 98,81 | 101 | 1791472 | 26986 | 32,23 |
| ERR3555308 | A797 | 0,87495 | 1919424 | 2200074 | 304 | Francisella | 99,78 | F. tularensis | 924 | 99,26 | 101 | 1795134 | 27711 | 32,24 |

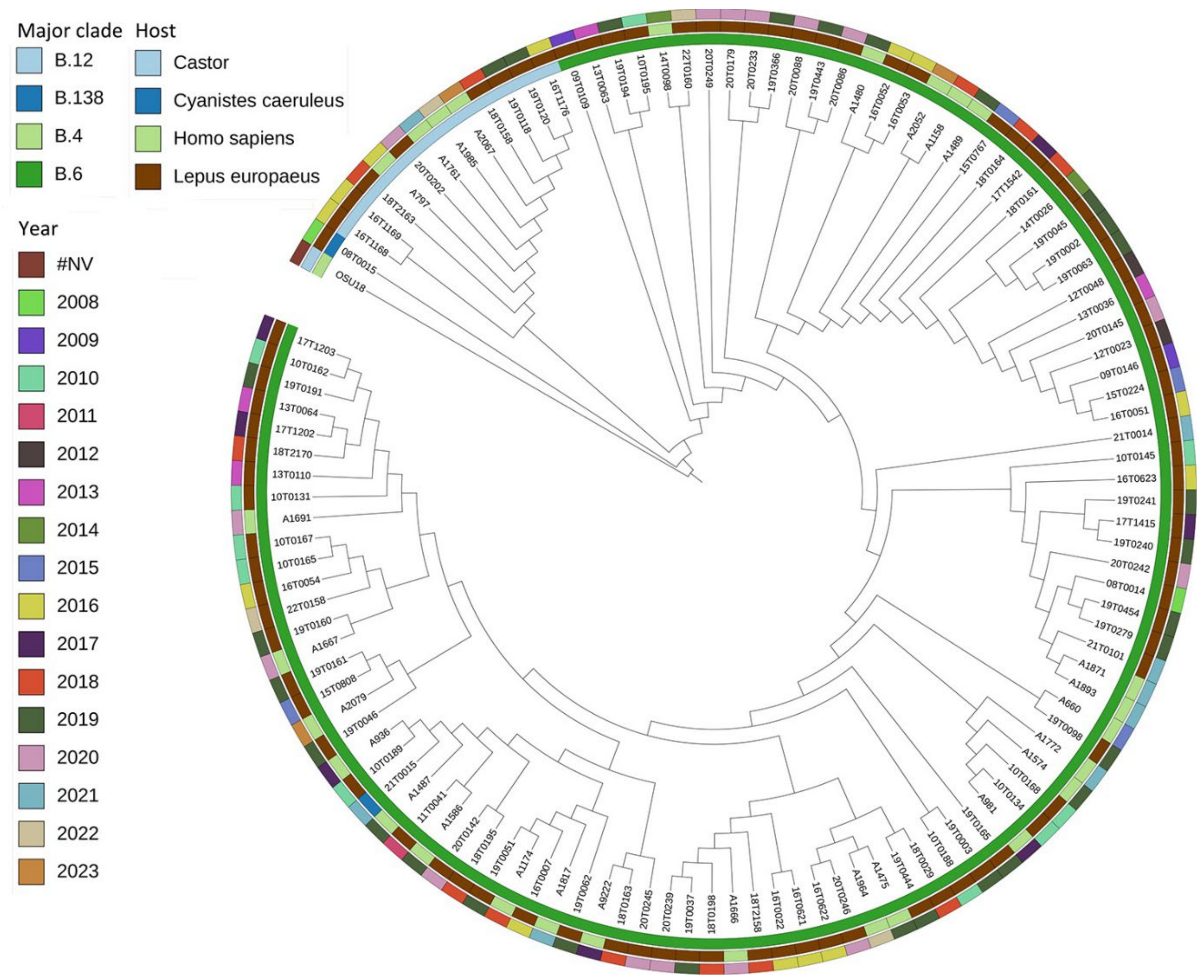


**Appendix Figure.** Phylogenetic tree of 115 analyzed strains of *Francisella tularensis* subsp. *holarctica* from *Baden*-Wuerttemberg.
